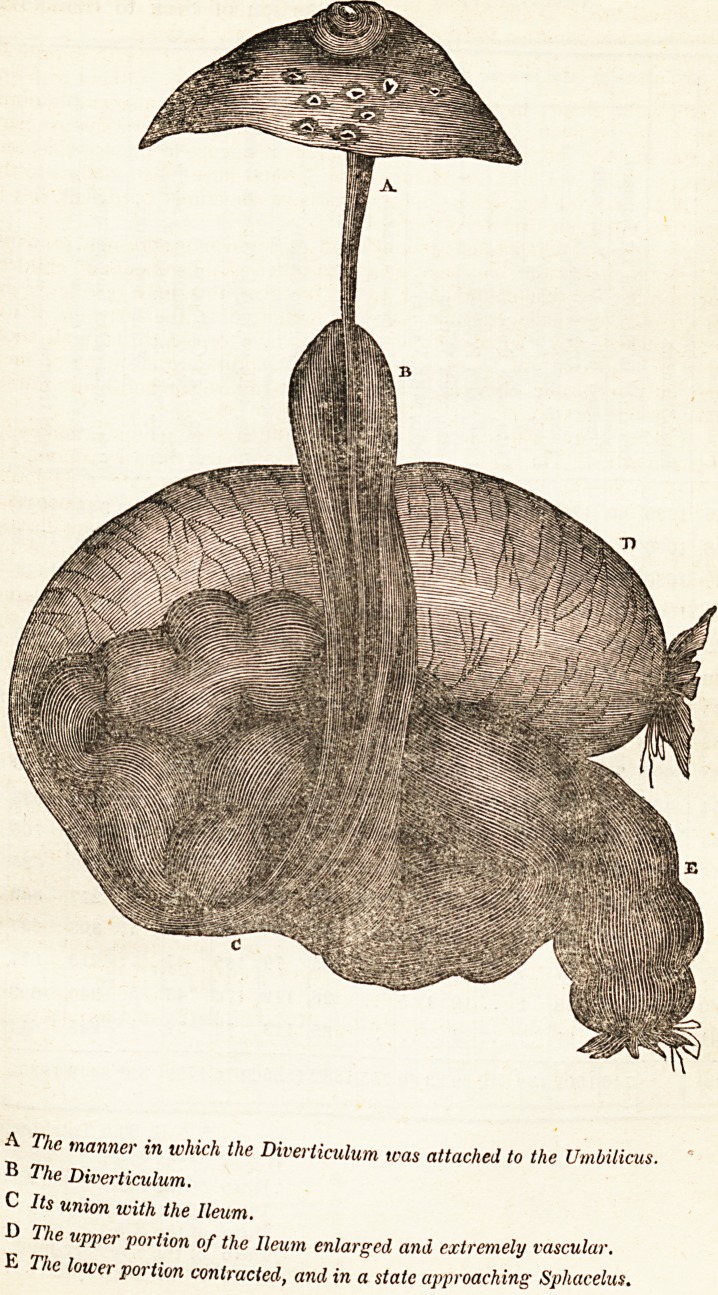# Extra-Limites

**Published:** 1842-07-01

**Authors:** 


### EXTRA-LIMITES.

A Statistical Account of the principal Diseases which have occurred amongst the Children admitted into the Royal Military Asylum, Chelsea, from the 1st of January, 1825, to the 31st of December, 1841, inclusive, with Remarks and Observations; together with the Detail of some peculiar Cases. By *Samuel Geo. Lawrance*, Surgeon to the Institution.

In offering the following statement of the principal diseases that have occurred amongst the children of the Royal Military Asylum, with a detail of some peculiar cases and *post-mortem* investigations, to the notice of my medical brethren, I am actuated by the desire of adding my contribution to medical statistics, as well as to pathological anatomy, both being now so much attended to, and properly appreciated.

Since my appointment to this Institution I have been in the habit of keeping notes of most of the diseases which have prevailed amongst the children, and, with very few exceptions, have instituted a post-mortem examination in every fatal case.

I hope, therefore, that the result, as it includes a period of seventeen years, will not be found devoid of interest.*

Since the year 1828 there has been a gradual reduction of the number in this Institution, which will be seen by referring to the following tabular return under the head "establishment," the number there stated being that which by order of Government it must not exceed. The number, however, must necessarily fluctuate very much, from the admissions not corresponding with the number leaving the Asylum as they gradually attain the prescribed age; this will be seen on reference to the return under the head "average daily strength of the establishment."

I shall now give the return stating the number of sick treated, and the fatal diseases which have occurred from the 1st Jan. 1825, to 31st Dec. 1841, inclusive, specifying the number that have had those complaints natural to children, as small-pox, measles, scarlet fever, chicken-pox, hooping-cough, &c. together with the average proportion of sick to strength, average of deaths to number of sick treated, &c. I ought, perhaps, to observe with respect to the return, that the

---

* As the nature and object of this Institution may not be generally known, I shall briefly state, that it was instituted by the late Duke of York, in the year 1803, for the maintenance and education of the children of soldiers of the regular army: the limited number at first was 1,000, which was afterwards increased until the year 1814, when the number amounted to 1,250 (850 boys, and 400 girls) and this was the maximum number at one time within its walls.

The children are admitted from the age of 5 to 10 years. The boys, when they attain the age of 14 years, if eligible, are enlisted as soldiers, but if not fit for the army are apprenticed to some trade, and the girls on their attaining the same age are sent out to service as domestic servants, &c.

In the year 1823 the female children were removed to the Royal Military Asylum at Southampton, a branch of this establishment, but which was abolished in November, 1840, when the few that remained (52) were sent here.

same boy may be admitted several times during the year for relapses, especially in cases of ophthalmia, porrigo, and in some other diseases, which make the numbers appear great under those particular heads. Without taking this into consideration it might give an erroneous idea as to the general amount of sickness amongst the children. Under the head ophthalmia all diseases of the eye are included; by far the most common are the various species of scrofulous ophthalmia in which also relapses are very frequent. No case of the purulent or Egyptian ophthalmia, once so prevalent here, has appeared for many years. Under the head "common fever," are included all kind of febrile affections not of a specific nature.

I shall now proceed with remarks and observations, detail of particular cases, and *post-mortem* examinations, in chronological order, intending them to form a sort of commentary on the return.

1825.

In the beginning of *April* of this year measles appeared among the children, and during that month 57 were admitted for this complaint, of which number 20 had it severely, attended with inflammation of the trachea and lungs, and three of them died, the remaining 37 had a comparatively mild disease.

In *June* scarlet fever broke out and continued until the end of September; during this period 49 children had this disease, but it was generally of a mild character, and dropsical symptoms supervened during convalescence only in one boy, and he recovered.

In the beginning of *October* measles again prevailed about a fortnight after the scarlet fever had ceased, and during this month and *November* 26 were attacked with it, of which number 12 were severe cases, and 14 mild—one of the former dying consumptive in the month of February of the following year.

*Seven* cases of small-pox occurred during the months of March, April, May, and June, of this year, one of which only was severe.

There were also seven cases of chicken-pox all very mild, with little fever, and which occurred at the same time that the small-pox prevailed.

In the month of September a boy, aged 13 years, had the operation of extirpation of the right eye performed on account of a cancerous fungus, from which he perfectly recovered.

Of the eight fatal cases which occurred this year I shall briefly detail the *post-mortem* examination of two, which I deem more particularly interesting.

*Case* 1.—Robert Lusk, aged 13 years, a boy of decided scrofulous diathesis, had been suffering for a considerable period from the usual symptoms of mesenteric disease. He became greatly emaciated and died on the 23rd Feb. 1825.

*Autopsy*, 48 hours after death.

*External appearance.*—Extreme emaciation of the body generally, the abdomen was much distended, but evidently not from air, it being very hard and incompressible.

*Thorax.*—The lungs on both sides of the chest partially adhered to the *pleuræ costalis* by long threads of false membrane. On cutting into their substance the structure of both was perfectly healthy.

*Pericardium* contained ℥ss. of serum.

*Heart* rather small.

*Abdomen.*—On making an attempt to cut into this part of the body, the whole contents—liver, omentum, stomach, intestines, &c. were perceived to be firmly adherent to the peritoneum, and consolidated into one mass.

The liver and stomach adhered so firmly to the under surface of the diaphragm that they could not be separated, even with a scalpel, without cutting into one or other organ. The large and small intestines were completely agglutinated together and to the contiguous viscera, forming with the greatly enlarged and indurated *mesenteric glands,* one confused mass of disease. The *peritoneum* was very much thickened and of a cartilaginous hardness. On opening the *stomach,* which was much distended with air, a small quantity of mucus and a dark-coloured fluid, resembling coffee-grounds, were seen. The liver was of its natural colour, but its texture was harder than usual. The intestines were much contracted, contained very little air, and were filled with a soft pultaceous fæcal matter of a light yellow colour.

The *mesenteric glands* were very greatly enlarged, and of a cheesy consistence.

Of the numerous fatal cases of tabes mesenterica which I have examined, none have exhibited so much general disease of the contents of the abdomen as the above, yet there was nothing very peculiar in the symptoms. He had no great degree of fever, pain was seldom complained of in the abdomen, unless under pressure, the tongue was in general clean, his appetite was very capricious. The pulse was unusually slow towards the latter stage of his complaint, there was almost constant diarrhœa, and he appeared ultimately to die from inanition.

*Case* 2.—Henry Williams, aged 14 years, of scrofulous habit, after acute suffering died with symptoms of peritoneal and intestinal inflammation, on the 15th May, 1825.

*Autopsy,* 40 hours after death.

*Thorax.*—The *right lung* was perfectly healthy, but the *left* partially adhered to the *pleura costalis,* was much hepatised, and contained several tubercles.

*Abdomen.*—On opening this cavity, a very extensive and confused mass of disease was exhibited. The transverse arch of the colon was ulcerated and communicated with the duodenum. All the convolutions of the *small intestines* were agglutinated together, and contained much liquid fæcal matter mixed with castor oil which had been swallowed. Several abscesses or purulent depôts had formed in various parts of the mesentery between the convolutions of the small intestines, and there were small ulcerated apertures at different places of the ileum, which communicated with these abscesses, and the intestines were so soft and altered in structure as to tear on the slightest force being used. The sigmoid flexure of the colon was ulcerated and loose fæcal matter had escaped into the pelvis behind the bladder.

The *peritoneum* was much thickened throughout. The *liver* adhered firmly to the diaphragm, stomach, and contiguous parts, but when cut into appeared of healthy structure. The gall-bladder contained a small quantity of bile. The *stomach* also adhered to the diaphragm and contiguous parts, its internal coat was healthy, and only contained a small quantity of a dark-coloured fluid.

This boy must have suffered from abdominal disease for a long time without complaining, for he had only been under treatment in the hospital three weeks.

His brother died in this Institution some years since of mesenteric disease.

1826.

In the month of January, a species of influenza or epidemic catarrh prevailed among the children, and 55 were admitted with it during this month, but the symptoms were mild, most of them recovering in a week or ten days.

In the month of May a boy, aged seven years, suffered amputation of the thigh, on account of scrofulous disease of the knee-joint, which did well.

During the Summer 12 children had the chicken-pox. All had very little fever.

In the Autumn measles appeared, and 20 had this disease—seven severely, two of whom died with symptoms of pneumonia—and in the remaining 13 the symptoms were mild.

There were also 12 attacked with hooping-cough; three of whom died, two with symptoms of pneumonia, and one from debility and gangrene of the cheek, apparently superinduced by the disease.

*Nine deaths* altogether occurred in this year, and it is worthy of remark that *eight* were from diseases of the respiratory organs. It should be mentioned that the Winter of this year was particularly severe, and the frost intense, the thermometer during the months of January and February being several times as low as 17° degrees of Fahrenheit, or 15° below the freezing point.

I shall give the *post-mortem* examination of one of those who died from hooping-cough.

*Case* 3.—Alfred Green, aged seven years, was attacked with hooping-cough on the 30th of October, and was a delicate child. The paroxysms of coughing were very violent, and he soon became much reduced and exhausted, emaciation rapidly came on, with sloughing and ulceration of the hips and lower part of the spine. About a week before his death, which took place on the 17th of December, he was attacked with sloughing ulceration of the gums on the *left side* of his mouth, and in a day or two a circular gangrenous spot, about the size of a shilling, appeared on the inside of the cheek of that side, and which on the day he died it had nearly perforated, the external part of the cheek having assumed the same inflamed and sloughy aspect.

*Autopsy,* 22 hours after death.

*Thorax.*—Complete and firm adhesion of the lungs on both sides of the chest to the ribs, requiring the scalpel and much force to separate them; the *pleura pulmonalis,* was also much thickened. On cutting into the substance of the lungs, the right appeared healthy, but the *left* was partially hepatised.

The *pericardium* contained about six ounces of serous fluid, the heart was natural.

*Abdomen.*—Nearly a quart of a turbid serous fluid was found in this cavity. The *omentum* had no adipose substance between its layers, but was much thickened and unusually vascular, and the lower part of it was so much indurated as to be of a cartilaginous consistence.

The *mesenteric glands* were much enlarged and indurated, several being as large as a moderate-sized walnut.

Of the two deaths from *phthisis pulmonalis* this year—one was a boy aged six years, in whom the disease appeared to have been excited by measles—and the other, aged 12 years, was (according to his mother's statement) the last of *twelve* of her children who had died of consumption. On the *post-mortem* examination both exhibited the usual appearance of tubercles, and vomicæ of various sizes in the lungs.

1827.

Five cases of scarlet fever occurred this year—two in the Spring and three in the Winter months—all severe, with much affection of the throat and fauces. There was a case of small-pox on the 28th of December, in a boy six years of age, who was said by his mother to have been vaccinated when *three weeks old.* A very indistinct and equivocal mark was observed on his admission into the Asylum in November, only one month previous to his being attacked with the small-pox. He had a severe and confluent form of the disease, leaving numerous permanent pits and marks on his face and body. It was also followed by several phlegmonous abscesses.

Four cases of chicken-pox also occurred this year.

Among the fatal cases there was one from pulmonary and mesenteric disease, having also caries of the shoulder-joint, which I shall relate.

*Case* 4.—William Lodge, aged seven years, of a highly scrofulous habit, was admitted into the hospital in November, 1826, for an abscess over the deltoid muscle of the left shoulder: he stated that it was caused by a severe blow he received on the part from a brush being thrown at him by another boy. It was in a few days punctured, and a large quantity of pus evacuated: soon after it was perceived that the abscess communicated with the joint; he was also suffering from symptoms of pulmonary and mesenteric disease. Hectic fever supervened, he gradually became much emaciated, and died on the 28th of April, 1827.

*Autopsy*, 36 hours after death.

*Thorax.*—The lungs on both sides of the chest adhered firmly to the *pleura costalis*, except a space on the right side, in which about six ounces of serous fluid were effused. On cutting into the substance of the lungs, both were found much diseased, being interspersed with tubercles and vomicæ.

The bronchial glands were much enlarged, and of a soft cheesy consistence.

*Pericardium* was thicker than usual, and contained nearly four ounces of serous fluid—*heart* natural.

*Abdomen.*—Upwards of a pint of turbid fluid mixed with flakes of lymph was found in this cavity. The *liver* was partially adherent to the diaphragm and side of the abdomen by threads of false membrane, and its peritoneal coat was much thickened. The *intestines* were of a pale colour, quite empty, and had a few spots resembling extravasated blood on their external surface.

The *mesenteric glands* were considerably enlarged and converted into a caseous consistence.

*Left Shoulder Joint.*—On examining this joint, the head of the *humerus* as well as the glenoid cavity of the scapula were found soft and carious. The caries of the humerus was confined to that part of the bone contained within the capsule of the joint, and in which was found a small quantity of curdy pus. Three external sinuses between the muscles communicated with the joint.

1828.

In the months of January and February of this year, scarlet fever prevailed, and sixteen had this disease, but it was of a mild character. There were also ten cases of chicken-pox. In the months of November and December *cynanche parotidea* was very prevalent among the children, and during those months thirty-seven had this complaint, from which it would appear to be a contagious disease, although I believe this is doubted by some. In several there was a great degree of fever, attended with much swelling of the parotid glands. I have never seen any instance of metastasis to the testes. The fatal cases were unusually numerous this year—the following were peculiar.

*Case* 5.—P. Field, aged seven years, was admitted into the hospital on 23rd January, 1828, with symptoms of typhus fever and affection of the chest—it might be termed typhoid pneumonia. There was early low delirium, great prostration of strength, and much dyspnœa, &c. He died on the 7th of February, fifteen days after his admission. During life it was remarked that the heart was felt pulsating on the *right* side of the chest; this could not be accounted for unless from original malformation, or from some large effusion of fluid on the left side, causing displacement by pressing the heart over to the other side: but there were no symptoms indicating any kind of effusion except a great degree of dyspnœa. And there was no malformation of the bones of the chest.* The *post-mortem* examination which I shall now relate will explain the cause.

* Sir Charles Bell, in his work on Anatomy (Vol. 1, page 435, Edit. 1811), mentions an instance of " a boy eight years of age, who had a great collection of matter in the chest, whose heart was so displaced by a vast quantity (no less than

*Thorax.*—On opening this cavity the lung of the left side, which was very much larger than natural, and consisted of *three* lobes, was seen to occupy the whole of the left side of the chest, and to extend over to the right, as far as the commencement of the cartilages of the ribs of that side, displacing the heart by pushing it entirely over to the right, which side it nearly occupied, as only a very small portion of the right lung was visible, it being under the heart, flattened and compressed on the ribs and spine, to which and the *pericardium* the whole of this lung firmly adhered, requiring considerable force to separate them. This lung had only two lobes. The *left lung* had a few slight adhesions to the *pleura costalis*. On making incisions into the substance of the lungs, *both* were found to be partially hepatized, particularly the left, which was very large and heavy, and portions of it thrown into water immediately sunk.

On opening the *pericardium*, which contained no fluid, the heart was seen with its apex pointing to the right side, and the arch of the aorta so turned that the descending aorta continued its course down the left side of the vertebræ, as in ordinary cases.

*Abdomen.*—All the viscera of this cavity were in their natural position. The *spleen* was of a much harder and firmer consistence than usual, and a portion of its thin edge was white and of a cartilaginous hardness. All the rest of the viscera were of healthy appearance.

*Case* 6.—William Holt, æt. nine years, a scrofulous and delicate child, had been suffering under symptoms of dropsy and anasarca, with œdematous puffiness of face, and swelling of arms and legs, for some time. He had no cough, but occasionally his respiration was much affected, having great difficulty of breathing. He only complained now and then of pain in the abdomen. He died quite suddenly, without any increase of symptoms indicating his death. He went to bed on the night of the 16th February, much in the same state as he had been for two or three weeks before—at six o'clock the following morning the nurse found him dead.

*Autopsy*, 30 hours after death.

*Head.*—The brain and its membranes were in a healthy state, and nothing morbid was observed.

*Thorax.*—The right lung adhered firmly to the ribs, and on cutting into its substance, several masses of calcareous matter were seen, the size of large peas—it was otherwise healthy.

The *left lung* had no preternatural adhesions, but contained several small vomicæ, and at the upper and posterior part there was a large mass of calcareous matter, equal in size to a small filbert; several smaller masses were also dispersed throughout its parenchymatous structure, this lung being much more generally diseased them the other.

The *pericardium* was thicker than usual, and contained upwards of a pint of serous fluid; its inner surface exhibited several vascular patches, and shewed evident marks of its having been inflamed. The *heart* was natural, but the right auricle and venæ cavæ were unusually full of dark grumous blood.

*Abdomen.*—There was about a pint of serous fluid effused in this cavity. The *liver* and *spleen* were of a bright red colour as if from increased vascularity, but their structure appeared healthy. The *mesenteric glands* were much enlarged. This is the only instance I have seen of so much calcareous matter in the lungs of a child, and I believe it is not a common appearance of disease in the lungs of young persons.

---

four pounds) of pus, that it beat strongly on the right side of the breast while his disease continued, and as soon as the pus was evacuated, the beating of the heart returned naturally to the left side."

*Case* 7.—William Hynes, æt. nine years, was admitted into the hospital for disease of the spine, Aug. 10th, 1826, which had gradually progressed, resisting all the usual modes of treatment by issues, &c. &c. Notwithstanding the great excurvation of the spine, and extent of the disease, he had no paralysis of the extremities until the last two or three weeks of his existence. His appetite and digestion were always very good, his great suffering was from the difficulty of respiration, and the position he was latterly obliged to keep, for he could only lie on the right side, and when sitting up in bed always kept one hand up to his head to support it, his chin being protruded forwards, and the occiput thrown back between the shoulders. At length extensive sloughing of the right hip took place from his constantly lying on it, and he died on the 29th May, 1828.

*Autopsy*, 27 hours after death.

*External State.*—General emaciation of the body and limbs. The upper dorsal vertebræ excurvated to nearly an acute angle, the ribs much compressed, so as to considerably diminish the capacity of the thorax. An extensive slough on the right hip.

*Thorax.*—The lungs on both sides adhered firmly to the *pleura costalis*, and required considerable force to detach them from the ribs and spine; in doing which a large abscess was accidentally opened situated over the spinal column, extending longitudinally down the thorax, the sac of which covered the space from the last cervical vertebra to the eleventh dorsal, thus including 12 vertebræ, and contained upwards of four ounces of thick curdy pus mixed with fragments of the carious bodies of the vertebræ. The sac of the abscess, which was of considerable thickness, did not extend laterally beyond where the ribs are articulated to the spine. The whole of the bodies of the vertebræ included in the abscess were in a softened and carious state, and the intervertebral substance completely gone.

At the posterior part of the *left lung* was an abscess containing about an ounce of purulent matter, and another smaller one was found in the upper part of the other lobe of the same lung; the lungs on both sides were full of tubercles. The *pericardium* and heart were healthy.

*Abdomen.*—With the exception of the *mesenteric glands*, which were slightly enlarged and indurated, nothing morbid was observed.

This boy was of a highly scrofulous habit, and his case is remarkable from the extent of the disease affecting twelve of the vertebræ, viz. from the last cervical to the 11th dorsal inclusive.

The fatal case of *hæmoptysis* occurred in a boy nine years of age. He was admitted into the hospital on the 20th of June for a dry tickling cough without expectoration. On the morning of the 25th June he suddenly spat up while coughing about ℥ij. of dark-coloured blood—at nine o'clock in the evening the hæmorrhage recurred, and he suddenly threw up a considerable quantity of blood, full a pint, and in a few minutes after expired. On the *post-mortem* examination, both lungs were found to contain tubercles and small vomicæ, and in the *left lung*, about two inches from the division of the bronchii, a large abscess was seen containing grumous blood and pus, making it quite evident from whence the hæmorrhage had proceeded. There was also in the cavity of the abscess a mass of calcareous matter nearly as large as a small filbert. The portion of lung forming the parietes of the abscess was indurated and hepatized.

*Case* 8.—John Sharkey, æt. 11 years, was admitted into hospital on the 6th July for rheumatic inflammation of the *right knee*—in a day or two after, the left became similarly affected, and then the right wrist-joint, attended with severe pain and constitutional fever. He was bled generally and locally—took vin. colchici, calomel and opium, &c., but although the swelling, tenderness, and pain of the knee and wrist-joints subsided, the constitutional symptoms became aggravated, and what is rather remarkable, he did not complain of any pain in his chest, nor had he any particular dyspnœa, but gradually sunk and died on the 16th of July, ten days after his admission into the hospital.

*Autopsy,* 40 hours after death.

*Thorax.*—Both lungs were perfectly healthy, the left having only some slight adhesions to the *pleura costalis.* The *pericardium* was much thickened and unusually vascular, particularly towards the base of the heart. On opening it full four ounces of sero-purulent fluid escaped, exhibiting the heart, which was of its natural size, completely encased with a thick coat of coagulated lymph, of a yellow colour, and having a honeycomb appearance. The inner surface of the *pericardium* had also many portions of lymph attached to it, connecting it by band-like processes to the heart. On removing a portion of the yellow coating of lymph from the heart the muscular substance was observed to be redder than usual, and the blood-vessels much injected. The inner coat of the *aorta* near the valves was also redder than natural.

*Abdomen.*—No morbid appearances whatever were observed here.

*Right Knee-joint.*—As the original seat of complaint was in this joint, it was examined. On cutting into it, about ℥iss. of thick yellow lymph was found effused, and the cartilaginous surfaces of the joint were much redder than usual; there was not however the least erosion of the cartilages of the joint.

The left knee-joint was also examined, and considerable redness and vascularity of the lining synovial membrane was observed, but it only contained a small quantity of the natural glairy synovial fluid.

There were five deaths from *hydrocephalus* this year, and all were scrofulous children.

In *three* the lungs were found to be much diseased, being partially hepatized and tubercular.

In *two* the thorax was not examined, but one of these had caries of the fibula, and the other caries of two of the lumbar vertebræ, for which complaint he was under treatment in the hospital, when suddenly symptoms of cerebral effusion appeared. I shall give the *post-mortem* appearances of this case.

*Case* 9.—*Autopsy* of E. Curd, aged nine years, 24 hours after death.

*Head.*—There was no unusual vascularity or turgescence of the vessels of the brain; on the contrary, the brain was paler than usual, and there was slight sub-arachnoid effusion. The lateral ventricles were much distended with a limpid serous fluid; nearly two ounces were collected; the brain here was very soft, and the *foramen morroianum* widely open.

*Thorax.*—The *left lung* was firmly and universally adherent to the *pleura costalis,* and when cut into was found much congested and gorged with blood, but free from tubercles. The *right lung* had only a few slight adhesions to the ribs, and was perfectly healthy in structure.

*Pericardium* and *heart* were natural.

*Abdomen.*—No morbid appearances whatever were observed in any of the viscera contained in this cavity. On removing the intestines, &c. two abscesses were perceived, one on each side of the two upper lumbar vertebræ; the one on the left side contained about an ounce of thick curdy purulent matter, that on the right a rather less quantity of similar fluid. The cysts were separate, no communication existing between them, and the bodies of the vertebræ, forming part of the boundary of the abscesses, were softened and carious; the disease, however, appeared to be in an early stage, for the front part of the vertebræ intervening between the two cysts had its natural appearance.

1829.

*Two* cases of small-pox occurred this year. One a boy of nine years of age, the other twelve years. The former had a confluent form of the disease, leaving a few slight marks on his face, and he had several small abscesses afterwards on his head, the scalp having been much covered with the small-pox pustules. The latter had a very mild and modified form of the disease.

There were seven fatal cases this year. The following from jaundice may be deemed interesting.

*Case* 10.—Robert Naylor, aged twelve years, was admitted into the hospital on the 5th of August with the symptoms of jaundice, constipated bowels, and great yellowness of the skin and eyes, &c. Notwithstanding a variety of purgatives, conjoined with calomel, the alvine evacuations always exhibited a white cretaceous appearance. Emetics were also employed. His pulse was of natural frequency, and only became slow and irregular when the cerebral attack came on, which was early on the morning of the 29th, when he was seized with sudden and violent delirium. He was bled, and leeches applied to the temples; he soon sunk into a state of coma, and died the next day, the 30th.

*Autopsy*, 32 hours after death.

The whole of the body externally was of an intense yellow colour.

*Head.*—The skull-cup adhered very firmly to the dura mater; the vessels of the brain were much injected, and the *membranes* of the brain were of a yellow colour and over the internal part of the bones of the cranium. The *lateral ventricles* contained about two drachms of a very yellow serous fluid.

*Thorax.*—The lungs and contents of this cavity were in a perfectly healthy state. The cartilages of the ribs were tinged of a deep yellow colour.

*Abdomen.*—The *stomach* was much contracted, and contained a quantity of dark-coloured fluid of tenacious consistence, similar to what he had vomited a short time before death. On removing this fluid several spots resembling ecchymosed blood were observed on its internal coat.

The *liver* was unusually small, of a yellowish colour, and much harder than natural. The gall-bladder was small, and contained a dark green or nearly black fluid, of very tenacious consistence, much resembling melted glue, so thick that the strongest compression of the gall-bladder could not force it through the *ductus choledochus*. On removing this tenacious fluid the internal coat of the gall-bladder was seen to be unusually vascular.

The *mesenteric glands* were of healthy appearance, but between the folds of the mesentery there were several ecchymosed spots. The *spleen* was natural, and both the *small* and *large intestines* exhibited nothing morbid.

Jaundice is by no means an uncommon complaint among the children—but the above, and one other case exceedingly similar, which occurred in a girl ten years of age, in the year 1820, have been the *only two that have terminated fatally*.

The girl was to all appearance getting better, when, two days prior to her death, she was suddenly seized with a convulsive fit, without any premonitory symptoms whatever, indicating any affection of the head, and immediately after she became comatose, and remained so until she died. On a *post-mortem* examination upwards of an ounce of a deep *yellow* serous fluid was found in the *lateral ventricles*, the vessels of the brain were much injected, and the substance of both the brain and cerebellum were unusually soft. There were also about four ounces of serum effused in the chest, and one ounce in the *pericardium*. The *lungs* and *heart* were healthy. The *liver* was much smaller than usual, harder, and of a light yellow or straw colour. The *gall-bladder* perfectly empty and flaccid. The *stomach* was much distended with air, and contained nearly two ounces of a blackish, almost inky, tenacious matter, which adhered closely to the internal coat of this organ, but when this was scraped away, nothing morbid was observed.

1830.

Five boys had small-pox this year, two of whom had it in a severe and confluent form, and three in a mild and modified form. I may here state that, as a precaution, every child on admission is examined to ascertain whether he has had cow or small-pox, and the statement of the parent or relative who brings the child is not deemed satisfactory unless confirmed by evident marks of one or other of these diseases; if none are observed the child is immediately vaccinated.

It is remarkable that when small-pox does occur, the cases are solitary, or few, never spreading to any extent, though the highly contagious nature of small-pox is very well known, and more than three-fourths have only vaccination for their protection. Is not this a strong proof of its prophylactic power?

It certainly would be very desirable, (if ever possible) to know how many years the preservative effect of vaccination remains. It appears to me that vaccination is often performed much too early, before the infant's constitution may be said to be formed, for instance when only a few weeks old: *three months* after birth would be a period more likely to be permanently beneficial, and the earliest I would recommend.

During the months of May and June measles prevailed, and 48 children had this disease. Of this number 22 had it severely, followed by diarrhœa and ophthalmia, and 26 had it in a mild form, and none proved fatal.

Among the fatal diseases of this year, the following may be considered interesting.

*Case* 11.—James Picken, aged 13 years, was brought to the hospital at one o'clock p.m. on the 18th March, vomiting blood copiously; the attack occurred suddenly, while marching with his company into the hall to his dinner. He expired in a few minutes after his arrival at the hospital, having vomited upwards of a pint of dark coloured frothy blood. He was constitutionally a very delicate child, had been in the hospital for an attack of hæmaturia in January, and was discharged well in February. He was not known to suffer frow any pulmonary complaint before the occurrence of the hæmorrhage, although frequently seen at the hospital on account of chilblains, from which he suffered much. He never had any complaint of the urinary organs before the attack of hæmaturia in January, and which soon got well under treatment.

*Autopsy*, 46 hours after death.

*Thorax.*—The lungs on both sides presented a healthy appearance, except being in a highly congested state; there was a trifling *adhesion* of the *left lung* to the *pleura costalis*, at the posterior part near the spine. The *trachea* was full of dark coloured blood; it was carefully opened, and the divisions of the bronchi traced into the lungs, which were found much congested and gorged with blood, but no particular part from which the hæmorrhage had proceeded could be distinguished, no cavity, cyst, or coagulum was seen. The pulmonary artery and veins were also carefully examined, but no lesion was detected. The *pericardium* and *heart* were natural, but both auricles and ventricles were quite empty, no blood or coagula being found in them.

*Abdomen.*—The *stomach* was distended with flatus, and was found to contain at least a pint of frothy grumous blood. On removing which, this organ was found to be perfectly healthy, The *liver* and large and small intestines exhibited a healthy appearance. On removing the latter the ureters were seen enormously enlarged, about half an inch in diameter; the point of the little finger could with ease be introduced into both these canals, and which were thus enlarged throughout their whole course to the bladder. The greater part of the glandular substance of the *right* kidney was wasted, and the pelvis formed a very large pouch or sac, and what little remained of the glandular substance was pale, soft, and flabby. The *left kidney* did not exhibit any morbid appearance, except a great enlargement of the pelvis and its ureter.

The bladder was strongly contracted, and contained about ℥iij. of straw-coloured urine; its internal surface was rather more vascular than natural.

It is rather extraordinary that so much disease of the kidneys and ureters should not have been made more manifest by symptoms during life.

The following is a remarkable case of a fatal obstruction and inflammation of the bowels caused by a preternatural pouch of the ileum, or *diverticulum ilii*, as it is termed by anatomists.

*Case* 12.—Edwin Mills, a stout healthy boy, 12 years of age, had been out on pass at Easter for a couple of days to see his mother. On his return here in the evening of the 11th April, he drank very copiously of cold pump-water, and went to bed. The following morning, at 9 o'clock, he was brought to the hospital, complaining of violent pain in his belly, particularly at the navel, with constant vomiting, and much general distension of the whole abdomen, which was also very painful when pressed upon. Pulse very small and quick; tongue covered with a yellowish fur; bowels constipated; countenance expressive of great anxiety and depression. Says he has not eaten anything likely to have disagreed with him, except a mince pie.

Bleeding, both general and topical, calomel and colocynth pills, purging enemata, fomentations, warm bath, blisters, &c. were successively employed without producing any beneficial effect. His pulse rapidly sunk and soon became imperceptible, the bowels remained obstinately obstructed, and the stomach constantly rejected everything he took. He died in thirty-six hours from the first attack. The symptoms were so similar to those of strangulated hernia, that it was suspected to be a case of intus-susception. The following *post-mortem* examination sufficiently explains the cause of death.

*Autopsy*, 38 hours after death.

*External appearance.*—The abdomen was excessively swollen and discoloured, of a dark green colour, with much general lividity of the whole body, and a great quantity of yellowish fluid had escaped from the mouth since death.

*Thorax.*—The lungs, heart, and pericardium were perfectly healthy.

*Abdomen.*—On opening this cavity about eight ounces of bloody serum flowed out. The *small intestines* were seen to be highly vascular, of a pinkish colour, encircled with numerous red vessels, and enormously distended, partly by air, but chiefly with a very large quantity of a turbid yellowish fluid, having a great number of small black currants floating in it. There must have been at least three quarts of this fluid in these intestines.

A singular appearance was now observed in the lower part of the abdomen. An adventitious portion of intestine in the form of a pouch, or cul-de-sac, somewhat resembling the finger of a glove, four inches in length and of a dark purple colour, was seen rising from among the convolutions of the ileum lying above the brim of the pelvis, and firmly attached at its upper or blind extremity to the umbilicus by a strong ligamentous cord, an inch and a half in length.

By tracing the intestines, the pouch was found to originate in the ileum, about fifteen inches from its termination in the *cæcum*, and this lower portion of the intestine was much contracted in diameter, of a livid purple colour, and only contained a very small quantity of bloody serum.

It now became evident that the convolutions of intestine which lay between the pouch and the cæcum, had got so compressed and strangulated that the canal was completely obstructed, for the livid colour commenced abruptly at the place where this pouch originated; and when the parts were *in situ* the ligamentous cord attached to the *umbilicus* was tense, and greatly on the stretch.

The *large intestines* did not exhibit any unusual vascularity.

The cæcum was not bound down closely to the iliacus internus muscle by the peritoneum, as usual, but a kind of meso-cæcum was formed, which allowed it to lay quite loose. It was much distended with air, and also the colon, both of which contained a large quantity of thick fluid resembling gruel, with oil floating in it, and apparently was part of the glysters which had been injected.

The *sigmoid flexure* of the *colon* and the *rectum* were small and much contracted. There were no solid fæces throughout the whole of the intestinal canal.

The *mesenteric vessels* were gorged with blood, particularly those of the lower strangulated portion of the ileum.

The *stomach* was greatly distended with air, it also contained above a pint of the same kind of turbid yellow fluid found in the small intestines, with numerous black currants in it. On removing the contents, a very slight redness only of the internal surface was observed.

All the remaining viscera were in a perfectly healthy state.

From the annexed drawing, taken at the time by a professional friend, who was present at the *post-mortem* examination, the peculiar appearance of the parts will perhaps be more accurately understood.

Anatomists have frequently noticed this kind of *lusus naturæ*, or preternatural pouch, occasionally found in the *intestinum ileum*, and which they have denominated *diverticulum ilii*. I have twice seen this occurrence in the bodies of children, who had died from another cause, quite unconneted with this lusus naturæ.

I have lately read in the first volume of the American Medical and Philosophical Register, published at New York in the year 1814, (which accidentally came into my hands,) a case in many respects very simliar to the one just narrated, but occurring in an adult.

As this publication is probably not much read in this country, I shall here transcribe the case.

*Case* 13.—" Case of Enteritis, accompanied with a preternatural formation of the Ileum. Communicated to the Editors of the American Medical and Philosophical Register, by John W. Francis, of *New York*, June 4th, 1810.

The writer of the following paper was an eye-witness to most of the facts which he relates. They are taken from memoranda made at the request of his preceptor, Dr. David Hosack, in whose practice the case occurred.

On the morning of December 22nd, 1809, Dr. Hosack was requested to visit a Captain D———, aged about thirty-five, of a slender habit of body, who was represented to be in an alarming condition. At the first view of the patient, it was perceived that he was afflicted with all the symptoms characteristic of enteritis, accompanied with those of ileus; viz., an acute and constant pain in the whole abdominal region, particularly about the umbilicus: the abdomen greatly distended, hard, and extremely sensible to the slightest touch, or whenever he attempted to move: vomiting of stercoraceous matter, and constipated state of the bowels; pulse small, tense, and frequent; respiration hurried and anxious; countenance livid; heat of the body increased somewhat beyond its natural temperature; and excessive thirst. These symptoms were attended with a great prostration of strength, and an extreme degree of restlessness.

Upon inquiring into the history of his complaint, it appeared that he had been first attacked while at the theatre on Wednesday evening, the 20th. On the morning of the day following, he was visited by an eminent physician, who directed an anti-spasmodic mixture, the symptoms of his disease being, at that time, slight. Deriving no relief from the medicine prescribed, Dr. Hosack was called upon on Friday morning, the 22nd, between the hours of eight and nine, when he found him labouring under all the symptoms above described.

From the best information that could be obtained, it was rendered higly probable that the exciting cause of his complaint was *cold*. He had been repeatedly subject to attacks of this kind, though less violent than the present, for several years past; at which times he was relieved by the ordinary method of treatment. Immediate recourse was now had to the lancet, and he lost blood to the amount of eighteen ounces.

A cathartic, composed of the pulv. jalap, and submuriate of quicksilver, each ten grains, was directed to be given, which was rejected in about an hour after he had taken it; and a similar one repeated with the same result. Blisters were applied near the umbilicus; fomentations of vinegar and water over the whole abdomen; and enemata of the oleum ricini and tinct. assafætid. were administered. These were partly discharged by vomiting; which afforded abundant proof that an inverted action of the whole intestinal canal had already taken place. In the afternoon the several applications to his surface were repeated; and during the remainder of the day he took, in divided doses, no less than two scruples of the submuriate of quicksilver, combined with opium and camphor; which, however, were rejected by vomiting shortly after they were taken. The enemata, rendered more active, were again given, but with no advantage.

At this time Dr. Miller visited the patient, in conjunction with Dr. Pott and Dr. Hosack. They united in recommending a continuance of the same mode of treatment that had been pursued.

In this condition he passed the night; the constipation of the bowels obstinately resisting every means used to obtain an evacuation.

On the morning of the 23rd, the submuriate of quicksilver, combined with opium, was again directed, in doses of 15 grains every two hours. The warm-bath was at the same time employed. It produced a temporary mitigation of his symptoms; but left him still more enfeebled.

His fate, which for some time had been probable, now became almost certain. The vomiting, which within the first thirty hours from the commencement of his disease had become stercoraceous, and which had continued with but little intermission to the present time, was now renewed. Attempts were made to allay it by the free use of opium, and other remedies usually indicated under similar circumstances.

The effect was an aggravation of all the symptoms. At 10 o'clock P. M. his dissolution was momentarily expected, his pulse scarcely perceptible, and his extremities cold.

He expired on Sunday morning, the 24th, at six o'clock, the vomiting having been incessant until about twenty minutes before his death.

*Morbid Appearances on Dissection.*—At two o'clock in the afternoon, the body was examined in the presence of the attending physicians, and several other professional characters.

The abdomen was tense and greatly distended: upon making a longitudinal incision into it, a considerable quantity of serous fluid issued out. Having completed the division, the intestines were found in a highly inflamed state, and of a dark red colour: the peritoneum lining the abdomen was also much inflamed, and covered with coagulable lymph. A remarkable deviation from the ordinary structure of the parts was now discovered to exist: a portion of intestine, attaching itself to the umbilicus, formed a union between it and a part of the intestinal canal. Upon further examination, this appendix was observed to be a *diverticulum* from the ileum. At the place of its union with the ileum it was enlarged and inflamed, in common with the upper portion of the small intestines; the remaining part was of a natural colour, and so intimately connected by its blind extremity at the umbilicus, as to leave little doubt of its being an original malformation. The ileum above this appendix was very much inflamed, extremely vascular, and in size equal to the transverse colon; while the lower portion was greatly contracted, and twisted round the diverticulum; and in this manner had been the means at least of aggravating, if not of inducing, the inflammation, and its consequences in this particular part of the intestinal canal. This portion of the ileum was of a dark, livid appearance, and had lost its tenacity. The great intestines were found completely emptied of their contents, and preternaturally contracted in their diameters throughout their course.

The *omentum, transverse colon,* and *stomach,* were at first altogether concealed by the distended state of the small intestines; and found in close contact with the diaphragm.

The *omentum* was irregularly drawn together. No unnatural appearance of the transverse colon was remarked.

The stomach lay in a circumscribed situation, was not more than two inches in width, and contracted in the same proportion throughout its whole extent. It was entirely empty; upon a minute inspection, no discolouration or affection of its coats were seen."

*Case* 14.—Samuel Tailby, aged 14 years, was admitted into the hospital on the 29th May, 1829, with pain in the left hip and limping, which he attributed to a fall he had down stairs several weeks before. It was soon perceived that there was incipient disease of the hip-joint from the lengthening of the limb and other symptoms. By means of rest, cupping, leeches, blisters, and an issue, the disease was apparently subdued, and at the end of September following he was so much better that he was allowed moderate exercise on crutches, but not to rest on the diseased limb. His general health at this time was very good, and the issue was nearly healed. He continued apparently well without any pain in his hip until the 5th February 1830, six months after all the symptoms had subsided, when he had a return of pain in the part—most probably from his using the limb too freely, it being very difficult to prevent him from walking more than he should have done, and he was frequently detected doing so without his crutches. In a few weeks an abscess formed in the front of the thigh; it was punctured, and much purulent matter evacuated; other abscesses formed, hectic fever supervened, and the disease proceeded with rapid strides, causing great disorganization of parts, to a fatal termination, which took place on the 4th June, 1830.

*Autopsy*, 26 hours after death.

*External appearance.*—Great emaciation of the arms and trunk of the body; both lower limbs were much swelled and œdematous, particularly the diseased one, and the œdema extended to the loins. The right hip was inflamed and ulcerated from the effect of pressure, and also the lower part of the spine. There were several ulcerated apertures and sinuses between the muscles of the left thigh, leading to the abscess in the hip-joint. The *femur* was dislocated on the dorsum of the ilium, and had ulcerated its way out, so that the *trochanter-major* was seen protruding through a large ulcer on the nates. The integuments of the nates and thighs were of a livid purple colour, and in a state of incipient gangrene. An erysipelatous inflammation had appeared on his thighs the day before his death, and the cuticle was extensively separated by infiltration of a serous fluid.

*Thorax.*—With the exception of the *lungs* having a few slight adhesions to the *pleura costalis*, the viscera of this cavity were in a healthy state.

*Abdomen.*—The *liver* was smaller than usual, of a pale yellow colour, and its substance softer than natural. No other morbid appearances were observed here.

*Left hip-joint.*—An extensive incision was made from the opening for the evacuation of the first abscess, which formed in front of the thigh towards the knee, and this exposed several sinuses among the muscles of the thigh communicating with the joint, and containing a fetid sero-purulent matter. The muscles of the thigh and hip were of a pale colour and of a soft pulpy consistence, apparently caused by the serous effusion and purulent matter. The capsule of the joint, was destroyed, and of the acetabulum, only a small portion was left, and that was in a complete state of caries, from which a sinus was discovered leading to a formation of purulent matter within the pelvis, between the *iliacus internus* muscle and the concavity of the ilium; the surface of that bone being also in a corroded state.

The *head of the thigh bone* was laying on the dorsum of the ilium completely carious, divested of all cartilage, soft and spongy, and the neck and shaft of the bone was partially corroded as far as the *trochanter-minor*—the *trochanter-major* had its natural appearance. The muscles of the nates immediately surrounding the diseased bones were in a sloughy and disorganized state.

1831.

Hooping-cough prevailed during the Winter months of this year. Twenty-three children had this disease, one of whom, a weak and puny child of six years of age, died from it. As there was a remarkable similarity to the case of Alfred Green, who died of this complaint in 1826, I shall transcribe it from my notes.

*Case* 15.—John Kennedy, aged six years, a puny and delicate child of scrofulous habit, was admitted into the hospital, with hooping-cough, on the 30th of October. The paroxysms of coughing were very severe attended with fever, causing great exhaustion. At length ulceration and gangrene of the mouth and cheek appeared, with petechiæ on the skin, and other symptoms of extreme debility. Wine and tonics were given, the cough having ceased for about a fortnight before his death, which occurred on the 16th December.

*Autopsy*, 28 hours after death.

*External appearance.*—Great general emaciation, the abdomen was speckled with small spots of petechiæ, above the clavicles, and on the sides of the neck were large ecchymosed purple spots or vibices. The greater part of the scalp was covered with small ulcerated spots, especially on the occiput. There were two sloughy ulcers at the angles of the mouth, with sloughing of the gums, and incipient gangrene of the left cheek.

*Thorax.*—The lower portion of both lungs were slightly adherent to the *pleura costalis.* On cutting into their substance, numerous small tubercles and vomicæ were seen, but none of the vomicæ exceeded the size of a pea.

*Pericardium* and *heart* were natural.

*Abdomen.*—The viscera of this cavity had a healthy appearance, but at three separate places intus-suscepted portions of the intestinum ileum were observed.

Among the other deaths which occurred this year the two following may be deemed worthy of notice. One from an abscess in the brain, and the other from rheumatic carditis.

*Case* 16.—Duncan M'Craig, aged eight years, was admitted into the Asylum on the 24th Oct. 1831, when he appeared to be in good health. On the following day he was brought to the hospital on account of refusing his food. He made no complaint but want of appetite, and had no febrile symptoms. Tongue clean; pulse natural. A purgative was given; and on the 26th he was dismissed, having no apparent complaint. On the 31st Oct. he was again brought to the hospital, still having no constitutional disturbance; but it was observed that he was inactive and dull, continuing to refuse his food; he now also complained of head-ache, particularly on the left side of his forehead; and at this part, about an inch above the superciliary ridge of the frontal bone, there was a small inflamed spot, somewhat resembling a small boil, from which a few drops of purulent matter were pressed out. On being questioned whether he could account for the pain in his head, he said he had had a fall in the street a week before his admission here, and struck the left side of his forehead against the ground; but the skin was only slightly bruised, and he neither felt sick at the time nor afterwards. His tongue was clean: bowels torpid. A dose of scammony and calomel was given him, and a bread poultice applied to the boil on his forehead. In three days the boil was healed, very little discharge having come from it, and only a small cicatrix remained. During this time he did not complain much of his head, and was walking about the ward.

No alteration took place until Nov. 5th, when febrile symptoms appeared; the tongue was slightly furred, skin hot, and increased pain in his forehead. Leeches were applied to the temples, a brisk cathartic of jalap prescribed, followed by saline medicine. There was nothing unusual in the alvine discharges. This was the first day that any serious affection of the head was indicated.

7th. The febrile symptoms were much moderated, and he said his head felt better; he dozed a great deal, and disliked to be disturbed, but was perfectly sensible, and the pupils of his eyes were neither unnaturally dilated nor contracted.

8th. This morning his pulse was found to be remarkably slow (60 in a minute); tongue more furred, and his bowels were torpid. He did not complain much of his head, but when he did he always pointed to the left side of his forehead. An emetic of ipecacuanha was prescribed, followed by calomel and rheubarb. At the evening visit, seven o'clock, the emetic had operated gently, but little was charged; and he had had two scanty alvine evacuations. Pulse 60, small, as in the morning; skin of natural temperature, and he appeared to be in a tranquil sleep. During the night the nurse, hearing him make a moaning noise, went to him; he was perfectly sensible, spoke to her, and said he did not want anything. At six o'clock the following morning (Nov. 9th) the nurse found him dying, and he soon after expired.

*Autopsy*, 29 hours after death.

*Head.*—On reflecting the scalp from the bone, particular attention was directed to the left side of the *os frontis*, where the boy had complained of most pain. A small carious perforation of the bone was there perceived, about an inch above the superciliary ridge, which would admit a small-sized probe; and the bone round this hole had a dull red appearance, apparently from increased vascularity. The internal surface of the scalp had a small dimple-like depression, corresponding to the hole in the bone, and the minute cicatrix on the skin of the forehead, left by the healing of the boil. The *calvarium* was now removed, and which adhered less strongly to the dura mater than is usual in young subjects. On the internal surface of the os frontis a small prolongation, about the thickness of a probe, was seen proceeding from the *dura mater* to the perforation in the bone, resembling a vein or vessel entering it; immediately above which was a small spot of ecchymosis on the brain, about the size of a sixpence. The dura mater was now reflected; no particular vascularity or turgescence of the vessels of the brain was observed; but the anterior lobe of the left hemisphere appeared of a straw, or greenish-yellow colour, evidently denoting the site of an abscess, with distinct fluctuation when pressed. The posterior lobes were of natural appearance, as well as the whole of the right hemisphere. The *right side* of the brain was now sliced down to the lateral ventricle; on opening which a small quantity of limpid fluid escaped. Attempting to do the same on the left side, the brain gave way, and about *two ounces* of bland inodorous pus gushed out, and the cyst of an abscess became apparent. The posterior part of the left lateral ventricle was also found filled with pus; but it was difficult to say whether it had existed there prior to death, or took place from the bursting of the cyst of the abscess during the dissection. The thinnest part of the cyst was at the anterior part, near the ecchymosed spot, just above the perforation in the frontal bone. The cavity of the cyst was vascular and of a dark red colour.

The *cerebellum* was natural. The *thorax* and *abdomen* were examined, and the viscera of both these cavities were in a healthy state; but there was a small *diverticulum ilii,* or preternatural pouch, three inches in length, proceeding from the intestinum ileum, at the distance of fourteen or fifteen inches from its termination in the cæcum.

The efforts of nature to make an outlet for the matter in this case are well worthy of observation. A little process or prolongation, like a duct, of the thickness of a probe, extends from the abscess and dura mater to the frontal bone, through which a perforation is made, by the action of the absorbents, to the integuments of the forehead, where a small boil forms and breaks, thus making a direct external opening, communicating with the internal abscess in the brain, which, consequently, may be said to have broken externally.

This must have been a chronic abscess of the brain, originating independently of the fall, although that accident may have accelerated the fatal termination, for it is scarcely probable that so large a collection of matter could have formed and made its way externally, in the manner above stated, in so short a space of time as from the date of the fall (about three weeks), and with so little constitutional r ritation.

His mother being questioned about his fall, corroborated the boy's statement, and said that it was only a slight cut or graze; she did not consider the hurt of any importance; but added, that he was always a very delicate child, not only as regarded his food, but in many other respects, and was possessed of great mental sensibility. Having lost his father about three months ago, he had fretted much on that account, and never was playful like other children subsequently to his father's death. With regard to the suddenness of his decease on the morning of the 9th November, may it not have been owing to the abscess having suddenly burst into the left lateral ventricle? for it is to be remarked, that there were no symptoms of effusion in the brain, no dilatation of the pupils, no coma, the boy being perfectly sensible to the latest period at which he was seen prior to death.

*Case* 17.—Chas Mahon, aged 14 years, was subject to acute rheumatism, and which had evidently produced hypertrophy of the heart. In appearance he was a remarkably fine stout and healthy looking boy. His heart was felt beating strongly against the ribs at all times and when otherwise apparently quite well. This inordinate action of the heart was first perceived in Jan. 1830. He was occasionally bled from the arm, and locally by leeches over the region of the heart, and kept on low diet, with amelioration of the symptoms—which were chiefly embarassed breathing and quick pulse. It was also observed that there was a remarkable difference in the strength of the pulse of the left arm compared to the right, being strong, full, and bounding in the former, and small, sharp and thrilling in the latter. From the 27th April, 1830, when he was dismissed from the hospital, until the 10th Dec. 1831, he remained apparently well, except the chronic disease of the heart. He was as much as possible restricted from using much exercise, and his respiration during this time was not particularly affected, he also became fat and muscular. On the 10th Dec. he was admitted for another attack of acute rheumatism. His wrists, knees, and ankles were affected in succession, and he had high inflammatory fever. He was copiously bled, and took calomel and opium, colchicum, &c. Under this treatment, in about a week, he apparently got much better, a relapse, however, took place, and the rheumatic inflammation attacked his heart, occasioning pain and excessive dyspnœa, occurring in severe paroxysms, threatening immediate suffocation—the disease having then left the joints. As for a long time he was known to have enlargement of the heart a fatal result was anticipated, and he expired suddenly on the evening of the 31st of Dec. 1831.

*Autopsy*, 64 hours after death.

*External appearance.*—The body presented a remarkably well made and symmetrical form, stout and muscular. The chest was ample, but a slight projection of the ribs on the left side was evident, over the situation of the heart. The left wrist and both ankles were slightly œdematous and puffy—the parts which were last affected with rheumatic inflammation.

*Thorax.*—The *lungs* on both sides were adherent by apparently recent prolongations of coagulated lymph to the *pleura costalis*, and about two ounces of bloody serum were effused on each side. The lungs at their posterior part were slightly emphysematous, but were not otherwise diseased in structure.

The *pericardium* was enormously distended, occupied a great part of the front of the chest, and before it was opened measured 7¾ inches across in a horizontal direction. It was perceived that the base of the heart adhered to the pericardium in such a way as to form two pouches, one on each side, which were full of fluid, and completely altering the usual form of this bag. The pericardium was now opened, and twelve ounces of bloody serum were found in it; its internal surface was of a dark red colour, and covered with papillæ-like projections and much thickened; there were also vascular productions proceeding from it to the heart, the muscular substance of which was covered with similar papillary granulations.

The *heart* was enormously enlarged, and highly vascular, and there was a pendulous fleshy projection of a cartilaginous hardness, nearly an inch long, at the upper part near the origin of the left auricle. On opening the cavities, the interior lining of the auricles and great blood-vessels were found of a deep red colour. The parietes of both ventricles were considerably thicker than usual. The heart with its investing pericardium, (after the evacuation of the fluid) was found to weigh 1lb. 13oz. avoirdupois. Now according to the average healthy standard weight of organs, as obtained from the Croonian Lectures for 1838, delivered by Dr. Clendinning, and published in the London Medical Gazette, the average weight of the heart of an adult male, above puberty, is calculated at only nine ounces avoirdupois. This may give some idea of the very large size of the heart in the case above detailed, which, including the pericardium, weighed 29 ounces!!

In the case of rheumatic inflammation of the heart, which occurred in 1828, there was no increase of its size, and although carditis has not unfrequently occurred among the children, and generally accompanied with rheumatism, I have never seen such an increase of the muscular substance as in the above instance.

1832.

There was one case of small-pox this year, a boy eight years of age, who had a mild and modified form of the disease.

In the month of May, a boy, aged eleven years, suffered amputation of the leg below knee, for scrofulous disease of the ankle-joint, which did well.

Nine deaths occurred this year; I shall give a brief detail of one of those who died of phthisis pulmonalis, in whom an abscess in the right lung had made its way through the diaphragm and appeared below the ribs near the spine.

*Case* 18.—John Murphy, aged eight years, a boy of a highly scrofulous habit, with deformity of the chest, it being smaller than natural, compressed at the sides, with the sternum projecting in front, forming what is termed a *chicken-breast*, had been in hospital for some considerable time with symptoms of pulmonary consumption. He was also subject to occasional convulsive fits, at which time, his face and lips were of a livid purple hue, and these were always relieved by a small bleeding from the arm. On the 19th November, for the first time, a small fluctuating tumor was perceived immediately below the ribs on the *right side*, about four inches from the spine. On coughing an impetus was distinctly felt in the part. The swelling gradually increased, without any discolouration of the skin, and descended towards the posterior part of the crista of the ilium, forming a large oblong fluctuating tumor. On the 9th December, he was seized with sudden and severe dyspnœa and great pain in the situation of the swelling, accompanied with a rapid, small pulse, and great anxiety.

A lancet was passed into the tumor, when nearly a pint of thick, fetid pus gushed out, and which, on his coughing, was jerked out the distance of several inches. He was much relieved by this operation, particularly in his respiration. Hectic fever, however, continued with rapidly increasing emaciation, and purulent matter, was spontaneously evacuated, in variable quantity, from the puncture until his death, which took place on the 8th February, he was then reduced to a mere skeleton.

It is worthy of remark, that there was no expectoration of any kind, until about a week prior to his death, when he began to expectorate purulent matter, mixed with blood.

*Autopsy*, 26 hours after death.

*Thorax.*—The left lung was firmly adherent to the ribs and the pericardium; but only a small portion of the superior part of the *right* lung was adherent to the upper part of the ribs, the cavity on this side of the chest contained nearly a pint of thick purulent matter; the *pleura costalis* was covered with a thick layer of coagulated lymph, and the lung of this side was broken down in shreds, ragged, and ulcerated.

It was then ascertained that the pus had perforated the diaphragm and descended behind the peritoneum downwards towards the pelvis, and communicated with the opening which had been made by the lancet.

The *left lung* when incised was found to be full of tubercles and vomicæ, scarcely any portion of it being of its natural structure, indeed it is surprising how respiration could have been performed, both lungs being so extensively diseased.

The *pericardium* adhered closely to the lungs and surrounding parts, and contained only a very small quantity of serous fluid. The *heart* was rather small, and on opening its cavities the *foramen ovale* was widely open, a large swan quill could easily be passed through it, and the ordinary valvular fold was so little developed, that it could scarcely have prevented some portion of venous blood from constantly passing into the left auricle.

*Abdomen.*—The *liver* adhered by prolongations of coagulated lymph, or false membrane, to the under surface of the diaphragm, but its substance was healthy.

Several of the *mesenteric glands* were much altered in structure; being of a cartilaginous hardness, but not much larger in size than natural—the rest of the viscera were normal.

1833.

Two cases of small-pox occurred this year; both were of a mild and modified form.

The Spring of this year was remarkable on account of an epidemic catarrh, or influenza, which prevailed generally all over England. It commenced among the children here on the 5th of April, and continued to prevail until the 11th of May; during this period 130 were admitted into the hospital for this complaint. The catarrhal symptoms were, in many instances, very severe at the commencement with much fever, delicate children suffered greatly, and were ill for some time, but a great many others were quite well in a week or ten days. Relapses also were not unfrequent.

The officers and servants of the Institution were not exempted from this epidemic, and nearly the whole of them were more or less affected by it.

With regard to the treatment—saline aperients, demulcent remedies for the cough and hoarseness, and blisters where there was much difficulty of repiration were employed, but, as in almost all cases, much languor and prostration of strength existed, venesection was rarely performed; leeches and blisters were, however, had recourse to, in those instances in which urgent symptoms resembling pneumonia appeared.

There were three deaths this year; the one caused by caries of the cervical vertebræ and abscess of the lungs, I consider worthy of record.

*Case* 19.—James Irving, aged 12 years, of a highly strumous habit, and having a narrow and deformed chest, was admitted into the hospital on the 3rd of

October, 1832, on account of pain and stiffness at the back of the neck, with enlarged lymphatic glands on each side, immediately below the mastoid processes. These went on to suppurate, and then general swelling of the posterior part of the neck took place, and gradually increased, notwithstanding leeches, cupping and blisters, were successively employed. At length, a deep collection of matter formed among the muscles at the back part of the neck, attended with great constitutional irritation. It became necessary to open the abscess, which discharged a thick curdy pus, and was found to communicate with the vertebræ. Other small abscesses formed and were opened, forming deep sinuses, from which a profuse discharge of curdy purulent matter issued. Hectic fever became established, and great emaciation ensued. About a fortnight before his death, a swelling was observed just above the right clavicle, which became prominent when he coughed, and which subsided when the cough ceased; it was soft to the touch, and could be easily pressed down below the clavicle. The impulse given to the hand placed over it, when he coughed, at once proclaimed it to be an abscess within the chest.

He was for months before his death unable to hold up his head without support, and was constantly in the habit of supporting it with one of his hands. His head was generally bent forward with his chin resting on the sternum. His appetite for food was very good until within a week or two of his death, he slept tolerably well, and did not complain of much pain, except when the formation of an abscess took place, had very little cough, and did not expectorate purulent matter, but died completely exhausted on the 12th December, 1833.

*Autopsy,* 36 hours after death.

*External appearance.*—Great emaciation of the body and limbs, a flattened and mal-formed chest. Much enlargement, and general thickening of the integuments at the upper and posterior part or nape of the neck, with three fistulous orifices leading to the uppermost cervical vertebræ.

Two extensive ulcerations on each side of the neck, in front, just above the clavicles.

*Thorax.*—The *right lung* was generally and firmly adherent to the *pleura costalis,* and adjacent parts, particularly at its upper part just below the first rib, and here a large abscess was found in the substance of the lung, sufficiently large to contain a hen's egg, full of sanio-purulent matter. On making further incisions, numerous vomicæ, and hard tubercles were seen throughout its substance.

The *left lung* had only a few slight adhesions to the ribs. On cutting into its substance several small tubercles were seen, but none in a state of suppuration.

*Abdomen.*—The *viscera* in this cavity were perfectly normal, except a few patches of small tubercles of the size of mustard seeds, dispersed on the external surface of the small intestines.

*Cervical Vertebræ.*—The whole of these vertebræ were removed, and on examination a considerable quantity of thick purulent matter was found within the *theca vertebralis.*

The first, second, and third vertebræ were in a complete state of caries, particularly the *atlas* and *dentata,* the cartilages on which the condyloid processes of the occipital bone rest were corroded, and the *processus dentatus* of the second vertebra was entirely destroyed by the caries. Tortuous sinuses, which opened externally, communicated with the three first vertebræ, to which the disease was confined, the remaining cervical vertebræ being in a natural state.

1834.

In the month of March of this year measles appeared among the children, and continued until the 5th of April; there were 48 cases, none of which proved fatal, although many were very severe. Immediately this complaint ceased scarlet fever broke out, the first case admitted being on the 4th of April. It continued to prevail during the Spring and Summer, until the 31st July, during which period 36 children had this disease. It re-appeared in November and December, two cases occurring in these months, and so carrying it on to the subsequent year. Of this number, 22 had *scarlatina mitis*, which in one boy was followed by general anasarca, but he recovered; and 16 had *scarlatina anginosa*, with considerable affection of the throat and fauces. One of these died in 57 hours after the attack, and being the only case of scarlet fever which has proved fatal out of 139 cases that have occurred within the period embraced in this Report, I shall give an account of it.

*Case* 20.—James Hawkins, aged 14 years, a boy of strumous habit, belonging to the band, was admitted into the hospital, June 5th, complaining of chilliness, nausea, sore throat, and headache. Countenance dejected and pallid. Tongue covered with a thick yellow fur. Tonsils inflamed, enlarged and covered with ash-coloured sloughs—pulse very quick and small. He was ordered an emetic followed by 3 grs. of calomel, and a rhubarb draught, which operated well. June 6th, has had a very restless night, and appears much worse this morning, being in a comatose state, and cannot be made to answer questions. He keeps his teeth closed, so that it is almost impossible to inspect the throat; but what can be seen of it, is covered with dark-coloured sloughs,—breathing rattling, and difficult—pulse rapid and small, 130 to 140. A faint scarlet eruption covers the chest and thighs, external swelling of the throat from enlargement of the lymphatic glands. Skin nearly of natural temperature. He will not take medicine. Wine, orange and lemon juice were put into his mouth, and the throat and fauces syringed as much as could be done with a solution of the chloride of soda. In the evening he was put into a warm bath for a few minutes, there being a disposition to convulsions, and four leeches were applied below each ear, there being much external swelling. He continues in the same comatose state. The eruption continues out, but is of a pale red colour.

*June 7th.*—Has had a restless night with much jactitation, he however appears more sensible, but cannot speak, and it is with the greatest difficulty that a teaspoonful of any fluid can be swallowed. *Sesqui-carbonate* of *ammonia* in camphor mixture was tried, and the *linem. ammoniæ fortius* applied round the throat. About two o'clock P.M. he was evidently sinking, and lay in a complete state of coma, extremities cold—pulse small, rapid and irregular, and died exactly at six o'clock, fifty-seven hours from his admission into the hospital.

*Autopsy*, 24 hours after death.

*Head.*—The *dura mater* and surface of the brain was very vascular, and numerous bloody points were seen on slicing the latter down to the ventricles, in which no fluid was found, but the lining of these cavities was much injected, and the *plexus choroides* unusually turgid. The sinuses of the brain were gorged with blood.

*Thorax.*—The *right lung* was partially adherent to the *pleura costalis*, but its structure was perfectly healthy. The *left lung* adhered firmly to the ribs and adjacent parts; but on cutting into its substance it was found healthy and crepitating.

*Pericardium* and *heart* normal. The tonsils and posterior fauces were in a sloughy state, quite black. The *trachea* was examined, its internal lining was more vascular than natural, but there was no deposition of mucus or coagulated lymph.

*Abdomen.*—The small intestines appeared much congested, and the arteries and veins which surround them were distinctly and beautifully exhibited. The liver healthy—gall-bladder much distended with dark green bile. The *mesenteric glands* were enormously enlarged, several being converted into a decided calcareous matter, and others were of a caseous consistence. This boy appeared never to rally from the stage of collapse caused by the contagious virus.

Incontinence of urine is a very common complaint among the children here, and difficult of cure, in some cases dribbling from them during the day, but in the greater number it only occurs at night when in bed.

For this complaint I have employed tonics, as quinine and steel, also tr. cantharides, blisters over the sacrum, cold-bathing, &c., with variable success, and in some instances I have found the occasional passing of a catheter or steel sound prove beneficial, perhaps fear may have some effect in the latter case, as the children have generally a great dislike or even dread of this operation. In some instances, however, all remedies have proved ineffectual, and time alone has effected a cure, the habit ceasing as they approach puberty.

*Case* 21.—Among the many boys brought to the hospital for this complaint, was one named J. Hunt, aged six years and a half, who was in the constant habit of wetting his bed at night, but the incontinence did not happen in the day time, although he made water rather more frequently than natural. He was a delicate puny child, and born in the East Indies. He suffered very little pain, and had no difficulty in passing his urine, so that it was not suspected that he had stone. After trying tonics, &c. for some time ineffectually, I passed a steel sound, and then discovered that he had a small calculus in his bladder. The urethra being unusually large for a child of his age, and suffering so little from his complaint, it occurred to me that the operation of lithotripsy might be tried, particularly as he was a very docile and tractable child.

On the 17th January, 1834, I consulted Baron Heurteloupe, who was at that time frequently performing that operation in London, as to its practicability in his case. He examined the boy, and was of opinion that, the stone being small, the operation might be successfully done, and in the most liberal manner offered to perform it gratuitously. As there was no urgency, the child appearing to suffer so little, the Baron recommended the previous frequent introduction of sounds into the urethra, for several weeks prior to his operating, in order to ascertain its capacity, and to familiarize the urethra to the presence of instruments. This was done by the almost daily introduction of elastic-gum and metallic bougies, until a steel sound (No. 12 size) could be introduced with facility. The bladder was also occasionally injected with warm water, and by this previous treatment the fears of the child were overcome.

On the 19th of March, Baron Heurteloupe performed the operation in the presence of several medical gentlemen. The stone was seized with great skill and celerity, and was crushed by a few blows of the hammer. The operation was over in about two or three minutes, and the child did not appear to suffer anything beyond the fear natural upon such an occasion. The same evening he passed with his urine much sand and several minute fragments of the stone. On the following day there was some inflammation and swelling of the meatus urinarius, with slight mucous discharge from the urethra, and several fragments of stone were seen sticking within the urethra about half an inch down the canal; by means of a small forceps these were easily extracted. He had slept well, and made no complaint. He continued to pass small fragments and sand until the 25th of March, when he complained of a want to make water, but could not void any. On introducing a catheter, it could not be passed beyond four or five inches, a fragment of the calculus being evidently impacted in the urethra. Baron Heurteloupe happened to call very soon after this occurred, and after some time, by injecting warm water and a little manipulation with sounds and catheters, succeeded in pushing back the fragment into the bladder, and then the urine flowed freely; but even on this occasion, when more force was used than during the whole of the treatment, not a drop of blood was passed, although it might have been expected, from the urethra being scratched or wounded by the fragment. The following day (the 26th) a *second operation* was performed, and the Baron laid hold of, and crushed, two or three remaining fragments in the bladder, in as short a time as in the first operation, and with as little pain to the child.

For three or four days afterwards, sand and minute portions of the stone were voided; after which his urine became quite clear, he ceased to wet his bed, and has continued to the present time, upwards of seven years since the operation, quite free from any urinary complaint. On analysing the fragments of the calculus, it appeared to be composed of the triple phosphate, and the amount of the *detritus* or dry fragments collected (exclusive of much sandy matter necessarily lost) weighed forty grains. I have subsequently passed both sounds and catheters into his bladder at various times, to ascertain if any fresh stone had formed, but have never discovered any. He is now a drummer in an infantry regiment.

It is worthy of remark, that the above is the only case of stone which has occurred in this Institution, according to the hospital records, since it was founded in 1803, up to the present time, yet upwards of 7000 children, of both sexes, between the age of five and ten years, have been admitted during that period.

1835.

With the beginning of this year scarlet fever again appeared : there were ten cases : only *three* had much affection and ulceration of the throat.

Of the fatal cases in this year I deem the following interesting from the extent of the visceral disease, which was discovered on the *post-mortem* examination.

*Case* 22.—William Shell, aged 14 years, of a highly scrofulous habit, had been for a long time suffering from the usual symptoms of *marasmus,* and his emaciation proceeded very rapidly. It was evident that there existed considerable organic visceral disease, and all remedies proving ineffectual, he died on the 28th January, 1835, in an extremely emaciated state!

*Autopsy,* 46 hours after death.

*Thorax.—Both lungs* were free from any unnatural adhesions, but on being cut into were found full of small hard grey coloured tubercles, of the size of mustard seeds, dispersed in their parenchymatous structure.

*Pericardium* and *heart* normal.

*Abdomen.*—Here great tubercular disease was seen. The *omentum* was devoid of fat, but was entirely covered with minute tubercles.

The *liver* was of a very large size, partially adherent by threads of false membrane to the inner surface of the lower ribs, and under surface of the diaphragm. It had a dark red, and mottled appearance, and on making incisions into its substance, several tubercles were found, some softened and containing purulent matter.

The *spleen* was enormously enlarged, full of tubercles both externally and imbedded in its substance, many of which were in a state of suppuration and ulceration, which, contrasted with the dark red colour of the spleen, exhibited a very curious red and yellow speckled appearance. Small patches of coagulated lymph were also deposited on its surface. When removed from the body it was found to weigh 12 *ounces avoidupois !* nearly three times the weight of the healthy spleen of an adult.

The small intestines were healthy; the sigmoid flexure of the colon was much contracted to the extent of several inches.

The *mesenteric glands* were very much enlarged, and several converted into a yellow caseous consistence peculiar to scrofula. The under surface of the diaphragm was nearly covered with patches or depositions of tubercular matter. About four ounces of serous fluid were found effused in the pelvis.

The other death from *tabes mesenterica* was a boy aged five years, born in

Jamaica, and admitted from that place into the Institution only two months prior to his death. On examination the liver was found much indurated, of a light brown or nutmeg colour, and containing tubercles, some in a softened and suppurating state. The *spleen* also contained many small tubercles, but was of its natural size.

In the fatal case of hydrocephalus both the lungs and spleen were found tuberculated, but the tubercles were small and quite in an incipient state. I consider hydrocephalus to be one of the fatal forms of scrofula, for I may here observe, that of seventeen *post-mortem* examinations that I have made of this disease, tubercles of the lungs, or organic disease of some of the abdominal viscera, were found in nine—and in three of the other eight cases the thorax and abdomen were not examined, my attention not being then drawn to the subject.

1836.

There were three cases of small-pox in the months of May and June, two were of the confluent kind and one was of a mild and modified form.

Three cases of chicken-pox occurred at the same period. Whenever small-pox has appeared, there have been generally during its continuance a few cases of chicken-pox.

Although varicella very much resembles mild small-pox on its first appearance, yet I think it has sufficient distinguishing characters, especially as the disease progresses.

I shall quote the opinions of a few eminent physicians regarding the diagnosis of the two diseases.

"The eruption of the chicken-pox comes on with very little fever preceding it, or with a fever of no determined duration. The pimples of the chicken-pox are formed into little vesicles or pustules more quickly than those of small-pox. The matter in these pustules remains fluid, and never acquires the colour or consistence of the pus which appears in the pustules of small-pox. The pustules of the chicken-pox are always, in three or four days from their first appearance, formed into crusts."—Cullen.

"Chicken-pox can in some instances be distinguished from the small-pox only by its quicker progress towards maturation, and the shorter duration of the pustules; a watery vesicle always appearing on the second or third day from the eruption, and the turn at the farthest taking place on the fifth."—Heberden, Med. Trans. of the Col. of Physicians, vol. 1, Art. 17. Dr. Heberden also states—"that in chicken-pox he never saw any person with so many as 300 pustules on the whole body; he also notices the early abrasion of the vesicles; their irregular and oblong form, the shrivelled or wrinkled state of those which remain entire on the third or fourth day, and the radiating furrows of others, which have had their apices closed by a slight incrustation; the general appearance of the small scabs on the fifth day, at which time the small-pox are not at the height of their suppuration—sufficiently distinguish the eruption of chicken-pox from the firm, durable, and slowly maturating pustules of small-pox."

Dr. E. J. Clarke says, "chicken-pox is to be distinguished from small-pox by the less degree of fever, by the eruption first appearing on the back, and its drying or desquamating on the fourth or fifth day."

Dr. Willan says—"*Small-pox pustules* on the first and second day of their eruption are small, hard, globular, red, and painful: the sensation of them to the touch, on passing the finger over them, is similar to that which one might conceive would be excited by the pressure of small round seeds under the cuticle."

"In chicken-pox almost every vesicle has on the first day a hard inflamed margin; but the sensation communicated to the finger in this case, is like that from a round seed flattened by pressure." Dr. Willan also remarks—"that, as the vesicles of the chicken-pox appear *in succession*, during three or four days, different vesicles will be at once in different states of progress: and if the whole eruption, on the face, breast, and limbs, be examined on the fifth or sixth days, every gradation of the progress of the vesicles will appear at the same time. But this circumstance cannot take place in the *slow and regulated progress* of the small-pox."

I have frequently had occasion to notice the correctness of this last observation of Dr. Willan.

There was no death from disease this year, only one from an accident. A boy fell from the top of the stairs on a stone pavement below, fracturing extensively all the bones of the left side of his head. The *dura mater* was lacerated and the whole of the left *parietal* bone driven into the brain. Notwithstanding the severe nature of the accident, he survived it 58 hours, but in an insensible state.

### 1837.

This year was ushered in by the appearance of an epidemic catarrh or influenza among the children.

During the month of January and beginning of February sixty cases were admitted into the hospital, the symptoms were nearly the same in all, and much resembled those of the epidemic catarrh in 1833.

The weakly and delicate children suffered most, and one of the cases of phthisis pulmonalis which happened this year was evidently hastened to its fatal termination by this influenza.

On reference to the Tabular Return it will be seen that an unusual number were admitted for cutaneous complaints—namely, 261 in the course of the year. A pruriginous papular eruption prevailed, and after being apparently cured in a few weeks recurred, the relapses being very frequent. Although sulphur was freely employed both externally and internally, yet in a great number of cases it failed to cure this eruption, and tepid baths, with milk diet, and saline cooling aperients, appeared to be more beneficial.

There were three deaths this year; two from *phthisis* were of the ordinary kind, and exhibited on the examination after death the usual appearances—tubercles and vomicæ. The one from *tabes mesenterica* also exhibited the common appearances, tubercular depositions on the small intestines with small corresponding ulcers of the internal mucous coat, great enlargement of the mesenteric glands, and conversion of their texture into a caseous matter.

### 1838.

There was one case of small-pox this year in a boy, aged 13 years, who had a confluent form of the disease.

Three cases of chicken-pox appeared at the same time.

Eleven cases of scarlet fever occurred this year, extending from the month of March until the middle of July. Of this number five had a severe disease, with much ulceration of the throat and tonsils, and six had it in a mild manner; in two of the latter it was followed by anasarca. I have generally found that dropsical affections more frequently succeed the milder attacks of this disease.

There were two deaths this year. One from hydrocephalus—a boy, five years of age, who on admission here on the 20th April, was evidently suffering from affection of the brain, and he died on the 2nd of May, having only lived 12 days after his admission into the Institution.

On the *post-mortem* examination the brain was found to be very vascular, and upwards of two ounces of serous fluid were found in the lateral ventricles. The brain was unusually soft.

The viscera of the *thorax* and *abdomen* were perfectly healthy.

The other fatal case from ascites was rather interesting, of which I shall give a brief detail.

*Case* 23.—William Maccauly, aged eight years, an orphan from Trinidad, was admitted into the institution on the 4th of June, at which time, he was suffering under symptoms of anasarca and dropsy. He had a large tumid abdomen, general œdematous swelling of lower extremities, puffiness of the face and eyelids, countenance pallid and waxy, and the skin of the whole body of a peculiar pale yellow colour. Pulse quick and small; tongue flabby, and covered with a yellowish fur; lips pale and bloodless; the person who brought him here from the West Indies said that the boy had suffered much at Trinidad from ague.

Mercurials, diuretics, hydriodate of potash, with tonics, &c. were administered without benefit, and he died on the 30th of July.

*Autopsy,* 36 hours after death.

*Thorax.*—Nearly a pint of serous fluid was effused on each side of the chest. Lungs of a pale colour, structure healthy, and quite free from any unnatural adhesions.

*Pericardium* contained about six ounces of serous fluid, the *heart* was small, pale, and flaccid.

*Abdomen.*—Upwards of a quart of clear serous fluid was effused into the general cavity. *Stomach* empty, and much distended with air: *liver* very much enlarged, of an orange-yellow colour, granulated throughout, and much indurated. Gall-bladder full of green bile. *Spleen* was healthy, and not larger than natural. *Mesenteric glands* normal. No other morbid appearances were observed.

An interesting and curious case of hemiplegia occurred this year, which I think worth relating.

*Case* 24.—On the 14th June, 1838, Frederick Middleton, aged nine years, a pale but stout boy of his age, having congenital deformity of the chest, being what is commonly called chicken-breasted, was brought to the hospital, at nine o'clock in the morning, with the following symptoms, having been quite well at bed-time last night. Extreme dyspnœa, panting for breath, the heart is seen beating violently, great anxiety of countenance, no pulse can be felt at the wrists, face pale and puffy, feet cold, upper part of the body of natural heat, vomiting of bilious fluid. Complains of no pain any where, only of great difficulty of breathing, with palpitation of the heart. Had immediately some hot wine and water, and a cordial mixture, with sesqui-carbonate of ammonia given him, while a warm bath was preparing, and a purgative enema was also injected. At 11 o'clock, after coming out of the bath, he was bled, but little more than an ounce could be obtained. Still no pulse at the wrists.

Imp. empl. canth. regioni cordis.

At seven P. M. the dyspnœa and palpitation of the heart continuing unabated, the following was prescribed:—

℞. Hydrarg. chloridi, Pulv. jacobi veri āā gr. ij. Conf. opii q. s. f. pil. 4tis horis sumenda.

℞. Magnes. sulph. ℥ss. Infusi sennæ, Mist. camph. āā ℥iss. Liq. ammon. acet. ℥i. Sp. ætheris nitric. ʒii. M. capt. ¼ 4tis horis.

*June* 15*th.*—Has passed a restless night, but respiration is improved, although still much hurried and quick: less anxiety and pallor of countenance: bowels have acted freely, loose bilious motions. Pulse can now be felt at the wrists, but is very small, quick, and indistinct. The saline mixture was continued with the omission of the magn. sulph. and ♏ xx. tr. scillæ was added: and the calomel pills were continued, substituting gr. ij. pulv. ipecac. c. for the James's powder.

*June* 16*th.*—Passed a better night; his respiration is easier, but still hurried, and the action of the heart continues inordinate. Pulse very small, quick, and thready. Face now rather flushed, skin hot and dry; carotids pulsate strongly. Tongue covered with a brownish fur.

Enema purg. c̄ Ol. ricini et magnes. sulph. āā ℥ss. et persist. in usu mist. et pil. calomel. ter die.

Seven P. M.—Respiration much easier. Pulse at the wrist more distinct, but continues very quick.

*June 17th*—Has had a good night, and appears better, respiration less hurried, action of the heart less violent and irregular, pulse 120, small. His diet is merely tea and bread and milk; slight cough.

At 9 o'clock this evening his breathing became suddenly more embarrassed, his face flushed, and the action of the heart more violent; tongue clean. Six leeches to be applied over the cardiac region.

℞. Hydrarg. chloridi gr. j. Pulv. Jacobi veri gr. iij. M. s. sd. haust. c̄ magn. sulph. ℥ss. cras mane sumendas.

*June 18th.*—Has had a tolerable night, but his breathing is still hurried and laborious; bowels freely open; pulse 120 to 130, and very small.

Mist. salin. ℥j. cum tr. digitalis ♏v. 4tis horis.

At 7 P.M. his breathing became much worse, and now the difficulty of respiration appears to occur in paroxysms, as, for some hours during the day, he breathed with tolerable facility. Has also a great degree of tenesmus this evening.

To have a starch enema, with ♏xij. liq. opii sed. Batt., Hirudines iv. regioni cordis. Empl. cantharid. inter scapulas; and the following draught:

℞. Liq. ammon. acet. ʒij. Sp. ætheris sulph. c̄ Tr. hyosciam. āā ♏xx. Mist. camphoræ ʒvj. M. f. haust.

*June 19th.*—Was tranquil, and slept a great deal during the night. Respiration much better, and performed with less difficulty, but is still rather quick; tenesmus abated. Pulse 120, small and irregular. Makes much urine.

7 P.M. appears easier; respiration more quiet.

*June 20th.*—Passed a tranquil night; respiration easy, and less quick; cough, with slight expectoration; pulse 110, irregular; tongue clean; abdomen rather distended.

Cont. Mist. salin. cum Tr. digitalis, et sumat haust. aper. cras mane.

*21st.*—A good night, and is much better; breathing quite free and easy; pulse 110; skin cool; has voided a very large quantity of urine during the night; bowels open.

Mist. salin. cum Tr. digitalis, ter die.

Makes no complaint.

*22nd.*—Had a good night, but about 8 o'clock this morning he became suddenly pale, faint and collapsed, with a cold clammy skin, weak but irregular pulse. Some hot wine and water was immediately given him, and a cordial mixture with carbonate of ammonia prescribed. He rallied in a few hours, and then a purgative enema was administered. When visited at 7 P.M. he was found to be completely hemiplegic, the right side of his body being paralysed; and he had also lost altogether the power of speech, but was perfectly sensible, putting out his tongue when required to do so, and by motions of his head, replied to inquiries as to whether he had any pain in his head or elsewhere. He signified that he was in no pain. *His respiration* also was *quite free and easy.*

*23rd.*—Has had a good night; passes his urine involuntarily; bowels rather torpid; pupils of eyes slightly dilated, pulse 100, soft and small; complete paralysis of right side.

Mist. purg. ad sedes.

7 P.M. His bowels have been freely opened since morning.

℞. Hydr. c̄ cretâ gr. iv. 4tis horis. Applic. empl. cantharid. nuchæ.

*26th.*—No material change; his gums are now tender; pulse 86; the incontinence of urine has ceased, and he voids it naturally and in large quantity. The blister on his neck to be kept open.

Rep. hydr. c̄ cretâ bis in die.

*July 1st.*—He continues in the same state. Pulse 84; gums are kept tender; appetite good; pupils are now of natural appearance and contract and dilate freely on the approach of light. The paralytic arm and leg are much colder than on the other side. Blistered surface of neck discharges freely.

Baln. tepid. hac. vespere.

15*th*.—He appears better, for he can now bend and extend the paralytic leg, but has no power whatever over the arm. He perfectly comprehends every thing that is said to him, and by motions of his head signifies his assent or dissent to questions asked him, for he cannot speak a single word; has no pain in his head, nor has ever complained of it: pulse 80, regular and of good strength; gums still tender; he is now allowed broth diet.

Capt. hydr. c̄ cretâ gr. iv. omni nocte, and a purgative occasionally.

℞. Infus. cascarillæ ℥iv. Ammon. sesquicarb. gr. xij. M. capt. ¼ bis quotidie.

*August* 1*st*.—Very little change since last report, except that his general health improves, and he now takes no medicine except what is necessary to regulate the state of the bowels. His appetite is very good. He can move the paralytic leg, but cannot rest upon it or walk; the right arm is quite powerless and he is still unable to speak a single word.

14*th*.—He continues slowly to improve; can now walk about the ward with the help of a stick, dragging the paralytic leg, and can for the first time articulate distinctly the words yes, no, and nurse, but has no use whatever of the paralytic arm. He is allowed the full diet of the Institution.

*Sept*. 14*th*.—He is gaining flesh, and can now walk tolerably well unassisted, but his arm is quite powerless, and he is unable to speak any other words than the monosyllables above-mentioned.

From this period, and during the whole of the Winter, there were such slight variations in his general health and paralytic state as not to require any particular notice. His speech being still limited to yes and no until the 14th April, 1839, when he was attacked with measles, at that time prevalent in the institution; he had the disease rather severely, which rendered him very weak and unable to walk, although he could walk and even run tolerably well before the attack of measles, with only slight dragging of the paralysed leg.

*On the* 9*th May* he was sent with some other scrofulous children to Herne Bay for the benefit of sea-air and bathing. While there he had so severe an attack of fever that the surgeon despaired of his recovery.

*On the* 31*st Oct*. he returned here much improved in general health and strength, quite fat, having a florid healthy countenance. He can now walk very well, and even run without any assistance, with very slight dragging of his right leg. The paralytic state of his arm is but little improved; he can lift it above his head, but has not the least use of the fore-arm, and his fingers are constantly bent towards the palm of the hand, unless when counteracted and kept straight by means of a splint and bandage. The temperature of the paralytic arm and leg continues lower than the other side, although enveloped in flannel. There is very little wasting of the palsied limbs. The pulse is very small, and scarcely to be felt at the wrist of the affected arm, while it is full and of good strength in the other. His speech is not at all improved, for he still can only articulate the monosyllables yes and no. His countenance is intelligent, and with the exception of the paralysis, he appears to enjoy perfect health; his bowels are always torpid, requiring the frequent use of aloetic pills or some other purgative.

It being considered that the establishing some drain or counter-irritation near the head might be worthy of trial, on the 19*th Nov*. a seton was passed in the nape of the neck.

*On the* 6*th Dec*. he had an attack of *cynanche parotidea*, with much fever; in about ten days the fever and swelling of the parotid glands disappeared.

On the 13*th Jan*. 1840, no benefit having been derived from the seton in his neck, it was withdrawn, and in short there has been no alteration in his paralytic state since his return from Herne Bay on the 31st of Oct. last year.

On the *5th May* he was again sent to Herne Bay, from which place he returned in Oct. in the same paralytic state, but otherwise in robust health. Being deemed incurable he was dismissed from the Institution, and taken home by his mother.

This boy was admitted into the Asylum in April 1836, and had been generally healthy, being very rarely in the hospital until the sudden attack of affection of the heart on the morning of the 14th June, 1838. I consider this attack to have been probably caused by sudden serous effusion into the pericardium, particularly as it was so much relieved by calomel and diuretics, and also by the large quantity of pale-coloured urine which he passed.

The attack of hemiplegia on the 22nd of June, so quickly following the subsidence of the dyspnœa and cardiac symptoms, and without any premonitory affection of the head, I am unable to account for, but consider it a very remarkable metastasis of disease. It is also worthy of remark that, since the attack of hemiplegia, he has had no recurrence of palpitation of the heart or any difficulty of respiration.

I have seen this boy several times since he has left the Institution, and though apparently in very good health, he continues unable to speak, and in the same deplorable state of helplessness.

1839.

During the months of March and April, measles prevailed extensively among the children, 35 had this disease, of which number ten had it severely, and 25 had the complaint in a mild form.

In June scarlet fever appeared, but there were altogether only seven cases, from June to September, when it disappeared; of this number three had *scarlatina anginosa*, with much affection of the throat, and four *scarlatina mitis*, with very little affection of the throat. There were five fatal cases this year. The two following may be considered worthy of notice, one from marasmus, the other from scrofulous disease of both kidneys. They were brothers, and both were admitted into the Institution from Gibraltar, the one aged eight years, the other twelve years; and I think them well-marked instances of the hereditary nature of scrofula and tubercular disease.

*Case* 25.—Alexander Grant, aged eight years, a puny and delicate child, born in Scotland, but came from Gibraltar to this Institution in July, 1837; was admitted into the hospital in February, 1839, with advanced symptoms of mesenteric disease. On the 19th of April he had the measles, at this time prevalent in the Institution, but in a mild form, and he suffered chiefly from the disease of his digestive organs, although it might, *by increasing his debility, have accelerated his death,* which took place on the 28th April.

*Autopsy,* 54 hours after death.

*Thorax.*—The *left lung* was firmly adherent to the *pleura costalis* and adjacent parts, requiring much force to separate it. On cutting into it numerous tubercles were seen, and it was much indurated and hepatized. The *right lung* was only partially adherent to the *pleura costalis,* this also contained tubercles, several of which were in the first stage of softening or suppuration; it was not indurated like the other lung, but crepitated under pressure like healthy lung. The *pericardium* contained about two ounces of serum—heart natural.

*Abdomen.*—The *liver* adhered firmly to the peritoneum, diaphragm and ribs, it was large, of a brown or nutmeg colour, and contained a few tubercles in a softened state. Both the large and small intestines were studded with grey coloured tubercles, and were partially adherent to the peritoneum, and to each other, by threads of coagulated lymph. The *mesenteric glands* were universally diseased, greatly enlarged, and several converted into the peculiar scrofulous, caseous substance. The spleen had a few minute tubercles on its external surface. The *omentum* and *peritoneum* were plentifully studded with small grey tubercles of the size of mustard seeds, and some as large as small peas. The kidneys and urinary organs were in a perfectly healthy state.

*Case* 26.—Joseph Grant, aged twelve years, born at Gibraltar, was admitted into the Institution in July, 1836. He was of a scrofulous habit, with a dry furfuraceous state of the skin, and subject to occasional swelling of the submaxillary and cervical glands; but rarely in hospital, except for trifling complaints of a few days' duration, until the 5th Februrry, 1839, when he was admitted for severe ulcerated chilblains. In the beginning of March he was attacked with fever, but did not complain of much local pain; nor was attention drawn to the state of his urinary organs until the 28th March, when he complained of pain and smarting in making water, with a frequent desire to make it. On examination, there was œdematous swelling of the prepuce, with slight enlargement of the body of the penis; and a small circumscribed swelling, of the size of a small hazel-nut, in the course of the urethra, just in front of the scrotum, very hard to the touch, and painful under pressure. Two or three of the inguinal glands were also enlarged, apparently from sympathetic irritation. He could assign no cause for this swelling, and said he had only perceived it a day or two. Pulse 120—a dry, unperspirable, and scurfy state of the skin; thirst; frequent micturition, and the general symptoms of fever were now present. His urine was observed to be turbid and milky, soon forming a deposit, and on being tested, had little or no effect on either litmus or turmeric paper. On the 30th of March the swelling had increased to the size of a small walnut, and fluctuation being now perceptible, it was punctured, and about a teaspoonful of purulent matter evacuated.

Mr. Stanley, surgeon of St. Bartholomew's Hospital, saw the boy with me at this time; and we sounded the bladder, suspecting calculus, but nothing could be perceived, except that it was in an extremely irritable state, and the operation appeared to cause much pain. In a few days under treatment the febrile symptoms were mitigated, but his pulse continued very quick, varying from 100 to 120; the irritable state of the bladder remained unmitigated, and the urine continued to exhibit the same turbid and milky appearance. He was frequently asked if he had any pain in his back or loins: he always said he had not. The region of the kidneys was often examined and strong pressure used, but he only complained of the continual desire to make water with pain in voiding it—passing only from half an ounce to an ounce at a time.

*April 6th*, the urine now passes both through the fistulous opening in front of the scrotum and orifice of the urethra: the meatus is also slightly ulcerated. The urine continues turbid and milky, and deposits a copious sediment very soon after it is voided. I took some of the urine to Dr. Prout, who was so kind as to analyse it. He said that it was serous and purulent, and that it was strumous pus, that it most probably proceeded from the kidneys, and that he had never seen such matter in calculous cases. He also prognosticated a fatal termination.

From this time there was little variation in the symptoms, only he gradually emaciated, and the character of the urine continued unchanged, but the purulent deposit varied as to quantity.

*May 6th.*—The urine now began to dribble from him when in the erect posture, and an accumulation of it appeared to take place in the perineum behind the scrotum, forming a small pouch in the membranous part of the urethra, and on pressing that part it oozed out from the fistulous opening and orifice of the urethra.

*May 16th.*—He now began to have evening febrile exacerbations and regular hectic fever commenced. He still says he has no pain any where except in the perineum and urethra, and chiefly suffers from the irritable state of the bladder, requiring him to make water almost every hour, and consequently disturbing his rest at night. There is also now more ulceration of the orifice of the urethra, and excoriation of the scrotum is threatened by the constant dribbling of the urine. 27th. The urine this morning contained an increased quantity of purulent deposit, mixed with ropy mucus, and his emaciation more sensibly and rapidly increases. The hectic fever continues, and his appetite, which has always been capricious, now begins to fail. The pouch or deposit of urine in the perineum does not enlarge, but on pressure is always found to contain a small quantity of urine.

*June 4th.*—To-day, for the first time, he complained of pain on the left side of his chest and over the region of the kidneys, increased by pressure. He has also had, for the last few days, a short dry cough; his countenance, at all times expressive of pain and anxiety, has now become more so, and he is evidently sinking under his disease. His pulse is very quick and wiry, and his appetite has entirely failed. 10th. He now speaks with difficulty, but is quite sensible. The stomach has become irritable and rejects both food and medicine. The irritability of the bladder is extreme, he is constantly passing small quantities of urine, which has uniformly preserved the same character, and on being kept for several days did not undergo decomposition or change.

The scrotum having been protected by oiled silk, has not excoriated, but there is deep ulceration round the *meatus urinarius.* His suffering is very great, and he is evidently dying. 11th. He died at 5 o'clock this afternoon, being perfectly sensible to the last. With regard to the treatment employed, it is not necessary to say anything as it could be only palliative. The peculiar, dry, furfuraceous state of the skin was remarkable, and a perspirable state of it could not be produced by any remedies.

*Autopsy*, 26 hours after death.

*Thorax.*—The *lungs* on both sides adhered to the parietes of the chest, but the adhesions were evidently of long standing. At the posterior part of both the right and left lung a large vomica was seen of the size of a filbert, containing purulent matter; a few tubercles were also found dispersed throughout their structure, the greater part of which however was healthy and crepitating. The *pericardium* and *heart* were natural.

*Abdomen.*—On opening this cavity the *omentum* and the whole of the peritoneal surface of the intestines and viscera were studded with small yellowish tubercles; also the folds of the mesentery, but the mesenteric glands were of natural size and appearance. The *right kidney* was next examined; on pressing it gently previous to incising it, pus flowed freely out through the divided ureter to the amount of two or three drachms. On cutting it open several abscesses were seen, and the pelvis was ulcerated, abraded, and entirely denuded of its mucous surface. On slitting open the canal of the ureter, the mucous lining was only partially ulcerated, there being small spots of ulceration on various parts of its internal surface. The *left kidney* also contained several abscesses, and, together with the ureter, exhibited the same appearance as the right. The *bladder*, which was very much contracted, was then removed, together with the penis and urethra. On slitting open the urethra its mucous surface was found abraded, and there were two ulcerated apertures in it from which urine and pus had evidently escaped during life, forming the small fistulous abscess which appeared in front of the scrotum. The *meatus urinarius* was also deeply ulcerated. The incision was then continued to the fundus of the bladder, which was quite empty, contracted, and rugous, its external muscular coat much thickened, and the internal completely denuded of its mucous surface. The *liver* and other viscera were normal.

It is curious to remark how the ulcerative process was continued throughout the whole of the urinary organs, from the kidneys to the orifice of the urethra.

1840.

There were ten cases of hooping-cough in the Spring of this year, five were very severe, one of which proved fatal.

A case of small-pox occurred on the 14th November in a boy aged 10 years, who was said to have been vaccinated, but as the mark was very indistinct and doubtful he was re-vaccinated here, but without success. He had a plentiful crop of pustules, but little fever, and no permanent marks were left. As usual, five cases of chicken-pox appeared about the same time.

The two cases of scarlet fever were slight, with very little affection of the throat.

Of the deaths this year, the case of hooping-cough, which proved fatal from a determination to the brain, is the only one worth relating.

*Case* 27.—William Crumpton, aged six years, was admitted into the hospital on the 7th March, 1840, for catarrhal fever, which soon merged into hooping-cough, at this time prevalent. He had violent paroxysms of coughing with great dyspnœa and evident affection of the head, being delirious the last few days of his life. He had leeches to the sternum and temples, blisters, saline and antimonial medicines, &c., but he progressively got worse, and died on the 21st March.

*Autopsy,* 46 hours after death.

*Head.*—Great difficulty was experienced in detaching the *calvarium,* it adhered so firmly to the *dura mater,* particularly at the posterior part. On slicing the brain down to the ventricles numerous bloody points appeared, *and all the vessels and sinuses of the brain were gorged with blood.* The lateral ventricles contained about one drachm of limpid fluid. The *plexus choroides* and the vessels which traverse the interior of these ventricles were much more conspicuous than usual. The substance of the brain was unusually firm. Nothing morbid besides the increased vascularity of the brain was observed.

*Thorax.*—On raising the sternum the *lungs* did not collapse, and were seen much distended with air and emphysematous, but there were no signs of inflammation, and no preternatural adhesions. They were of the natural bluish-grey colour: on cutting into their substance, which was very light and spongy, a great number of small granules or incipient tubercles were discovered.

The *trachea* and bronchial tubes were examined, but no unusual vascularity was observed; they only contained a small quantity of frothy mucus.

The *pericardium* and *heart* were natural. The abdomen was also examined, but no morbid appearances were observed.

1841.

No death occurred among the boys this year, but two girls died, one aged seven years, from a sudden hæmorrhage from the lungs, and the other, aged 14 years, of phthisis pulmonalis. I shall relate the case of the former as an example of the occasional termination of tubercular disease of the lungs.

*Case* 28.—Emily Evans, æt. seven years, was born on board-ship, on her parents' voyage to the West Indies, in January 1834. Both of them subsequently dying there, she was sent home from Demerara to the Royal Military Asylum, Southampton, in August 1838; and, on the reduction of that establishment in Nov. 1840, was transferred here. In March, 1841, she had a severe attack of jaundice, of which she was cured in a few weeks, but still evident symptoms of organic visceral disease remained—these were an irregular and torpid state of the bowels, a hard tumid abdomen, sallow countenance, constant quick pulse, capricious appetite, and gradual emaciation. She had very little cough, and without any expectoration, and did not complain of pain, except occasionally in the abdomen.

She was not confined to bed, and was able to take moderate exercise in the play-ground. On the evening of the 12th of August she went to bed apparently in the same state as she had been for some weeks previously. At six o'clock the following morning the nurse found her dead in bed surrounded with a large quantity of blood, which had evidently come from her mouth by vomiting.

*Autopsy*, 30 hours after death.

*Thorax.*—Slight adhesion of the inferior and posterior part of the left lung to the ribs; the right lung had no preternatural adhesions. *Both* lungs when incised exhibited numerous hard, grey, miliary tubercles, dispersed throughout their parenchymatous structure, but no vomicæ. In the middle lobe of the *right lung* a large jagged excavation was seen, containing a considerable quantity of grumous blood, but no purulent matter. It was quite evident that from this portion of lung the fatal hæmorrhage had originated.

The *pericardium* and *heart* were normal.

*Abdomen.*—The *liver* was of a pale yellow colour, and much harder than natural. Numerous small tubercles of a yellow colour were observed on its external peritoneal surface, particularly towards its thin edge, and similar tubercles were also found to pervade its substance.

The *spleen* was studded with tubercles both externally and in its internal structure.

The *small intestines* had several tubercular deposits on the peritoneal surface, and on slitting them open, corresponding ulcerations of the internal mucous coat were seen. The *mesenteric glands* were much enlarged and indurated, and the folds of the mesentery were studded with small, round, yellowish tubercles.

In the girl who died of phthisis pulmonalis, on a *post-mortem* examination, *both lungs* were found to contain tubercles and vomicæ, and in the upper and posterior part of the left lung there was an extensive abscess.

### Concluding Remarks.

Scarlet fever is frequently a very fatal disease, particularly to children, yet it will be seen, that within the period comprised in this statistical account, out of 139 treated, only one case proved fatal. I consider this fortunate result to be chiefly owing to the prompt medical assistance afforded, and the treatment not being interfered with by the fears and prejudices of parents and relations. How frequently is the call for medical aid deferred until the disease has gained an ascendancy which the most skilful employment of remedies cannot afterwards overcome.

In *scarlatina anginosa* I am convinced that early medical treatment is of the highest importance. I think it right to mention that I have found cold affusion or sponging with vinegar and water, according to circumstances and the season of the year, very beneficial. I do not find the children much frightened at the cold affusion as employed here, which is in the following manner.

The child, when covered with the scarlet eruption and the skin very hot and dry, is made to sit on a small stool placed in the middle of a large washing-tub, when about a gallon of cold water is quickly poured over him, he is then wiped dry and replaced in bed. This is in most cases followed by sleep and an abatement of the heat of skin and fever. I have never seen any harm result from this treatment, but it should be employed in the early stage of the disease.

It is useful to have the vapour of boiling vinegar dispersed through the ward or apartment, and for this purpose we use an earthenware apparatus of a conical shape, with a lamp, &c. which is easily procured in London.

I have before observed that the œdematous and dropsical affections which occasionally follow scarlet fever during the state of convalescence, more frequently occur after the milder attacks of this disease.

In an account of the scarlet fever which occurred among the children in

George Heriot's Hospital, Edinburgh, in the Winter of 1832-33, given by Mr. Wood, surgeon of that institution, and published in the Edinburgh Medical and Surgical Journal of January, 1835—he states "that out of 44 patients between the ages of 8 and 14 years, *nine* were afterwards affected with dropsical swellings." A much greater proportion than has ever occurred here; but he corroborates my observation by stating "that the patients who became affected with dropsical symptoms were not those who had been most severely ill of the primary fever."

Mr. Wood also says, that of the 44 boys who were affected with scarlet fever, "*five* were supposed to have had it previously—but the information procured on this subject is vague and unsatisfactory."

I have never seen an instance in this institution of scarlet fever occurring twice in the same individual.

As scarlet fever is highly contagious, it is here an invariable rule not to permit the convalescents to mix with the other children for a month, and not until *all desquamation of the skin* has entirely disappeared. Tepid baths are used occasionally during convalescence, in order to restore the healthy functions of the skin. Great care is also taken that the return to the usual full or animal diet should be gradual.

*Measles.*—The season of the year at which this disease appears makes a considerable difference in its severity, being most severe in Spring and Winter. Since the year 1825 there have been 240 cases of this disease, of which number *five* proved fatal. On the *post-mortem* examination *two* exhibited tracheal inflammation only, and *three* inflammation of the lungs. Three died in April, 1825, and two in the months of November and December, 1826.

*Hooping-Cough* is a disease not generally under much control, it will last a certain time under any treatment, but it can be much alleviated and rendered safe by medicine.

The treatment I employ consists of emetics, aperients, tepid baths, and a regulated diet, according as there may be more or less febrile excitement or tendency to inflammation of the lungs. Ipecacuanha, either the wine or powder, in very small doses, I have found very useful. I think also stimulant and antispasmodic embrocations serviceable.

This complaint does not appear to be so frequent at this institution as the other diseases of childhood. There have been only 69 cases within the period comprised in the preceding account, *five* of which proved fatal, two having died from the debilitating effects produced by the disease, two from inflammation of the lungs, and one from inflammation of the brain.

*Small-pox.*—It will be seen by the tabular return annexed, that there have been only 23 cases of this disease in 17 years, all of which, with the exception of four, have been subsequent to vaccination. Seven of them were severe and of the confluent kind, but in no instance was danger of a fatal result apprehended, there being no secondary fever on the maturation of the pustules. It is also worthy of remark that, notwithstanding the highly contagious nature of this complaint, it has never spread to any great extent among the children, although about three-fourths have only vaccination for their protection.

*Chicken-Pox.*—Of this complaint there have been 60 cases; it generally appeared at the same period with small-pox, but sometimes it prevailed alone.

*Epidemic Catarrh.*—The children suffered from this complaint in the years 1826—33—and 37, like the rest of the community, when it was prevalent in

London.* I think it however worthy of notice, that in the year 1832, when the Asiatic cholera prevailed so much in London and the suburbs, no instance of it occurred among the children or other inmates of this institution.

The only precaution taken was to prevent the children from going out of the building to visit their friends, as is customary at other times, nor were their friends permitted to come here while this disease was prevailing.

*Common Fevers.*—By this I intend to denote the numerous ephemeral and slight febrile affections to which children are subject, arising from cold, or from a loaded state of the alimentary canal, biliary derangements, &c. exclusive of those of a specific nature.

*Cutaneous Diseases.*—Those of most common occurrence here are prurigo, scabies, psoriasis, herpetic eruptions, and especially the various forms of porrigo capitis, not only the most frequent, but the most troublesome and intractable to which children are subject. Formerly, when 1250 children were in this Asylum, upwards of 100 have been affected with it at one time.

I have in vain sought for some specific or general application, but have found that the ointments and lotions which proved beneficial in some cases, have been completely unsuccessful in others. Without reference to the different names given by authors on cutaneous diseases to the various forms of porrigo, I am guided in the external treatment by the different stages of the disease, and the appearances which the scalp presents, which for practical purposes I thus classify:—1st, the inflammatory and pustular—2d, the humid and discharging—3d, the scabbing, dry, or furfuraceous stage.

In the first, I employ cataplasms of bread, lotions of thin gruel, decoction of poppies, with a small quantity of the liq. plumb. diacet., solutions of borax, &c. —all the hair is directed to be cut off, and the head to be shaved when it is in a state to bear it. In the second, where there is an ichorous discharge and an excoriated state of the scalp, the following lotion has been found useful:— ℞. Zinci oxyd. alb. ʒij. Mist. acaciæ, Aquæ, āā ℥j. M. The powder is insoluble, but when used the mixture is to be well shaken and applied to the excoriated places by means of a small piece of lint or camel's-hair pencil. The powder is deposited, checks the discharge, and after a few applications, scabbing or a dry state of the scalp is produced.

In the third stage, the use of bread poultices and emollient applications are again necessary to rèmove the encrustations. The *oleum sulphuretum* applied by a pencil brush sometimes also does this very well. Now various ointments are used, taking care that they are not too stimulating—as the ung. hydr. nitr., u. hyd. ammonio-chloridi, u. sulphuris, u. picis, &c., much diluted with ung. cetacei, suiting their strength according to the appearance the head exhibits during their use. And the above ointments are also often advantageously combined. Sometimes fluid applications seem to agree better than unguents, as lotions of diluted spirits of wine and acetic acid, a weak solution of argenti nitras or cupri sulphas, and lotions made with the sulphuret of potash, &c.

There have been a few cases of the peculiar species denominated *porrigo decalvans*, and they have generally been cured by the assiduous application of stimulating liniments. This complaint occurs among the healthy as well as the puny and delicate children, the internal or constitutional treatment therefore must vary accordingly. In the former, a regulated diet and the occasional use of purgatives is all that is required; but in the latter, purgatives, alteratives, and tonics, are necessary, for in a great many cases the digestive organs are much in fault.

---

* For the symptoms and character of which, the reader is referred to the annual remarks and observations of those years.

The alvine evacuations being morbid and unhealthy, a cachectic or scrofulous habit prevailing, this must of course be corrected before we can expect to derive benefit from any external treatment.

*Diet* is also a most important point, quite as much as the medical treatment; for if full animal diet be given to gross and plethoric children, the complaint will baffle all our remedial measures, and not be easily subdued: and vice versâ, in pale, scrofulous, and puny children, a meagre diet is equally bad. I think it right to state, that in this respect my experience and observation very much correspond with the treatment recommended by Mr. Macilwain in his valuable little work on this disease, entitled, "Clinical Observations on the Constitutional Origin of the various Forms of Porrigo, &c. &c. By George Macilwain. Lond. 1833." But I think he undervalues the use of topical applications, for though a regulated diet and attention to the state of the bowels and biliary secretion is absolutely necessary, yet external remedies most assuredly will expedite the cure. I have found that this complaint prevails most in Spring and Summer, and requires the utmost care and attention to prevent its spreading; it also more particularly affects the younger children, or those under nine years of age—very few, comparatively, above that age, being afflicted with it. Relapses are frequent, especially where there has been a want of cleanliness. That it occurs in some children spontaneously I am well convinced, especially in delicate and scrofulous children. I have also seen it appear during convalescence in those who have been debilitated by an attack of some febrile complaint, while in hospital, and removed from all source of contagion.

It is often impossible to predict the length of time that may be required to effect a cure of this complaint, many getting well in a few weeks, others not for several months or even years, including relapses, which are very frequent. A return to an unsuitable diet and inattention to cleanliness are the common causes of a return of the disorder. Some children are evidently more prone to this disease than others, and I have remarked that those having red hair are generally difficult to cure. At present (Dec. 1841) there are eleven cases of porrigo under treatment, five only of which are inveterate and of long standing.

I have already mentioned that diet is of much importance in the cure of this complaint, but it is very difficult in large establishments of children, of different ages, to form a diet suitable to each, both as to quantity and quality, with a due proportion of animal and vegetable food. The Diet Table of this Institution is as follows:—

DIET TABLE, ROYAL MILITARY ASYLUM, FOR ONE CHILD,

| DAYS. | BREAKFAST. | DINNER. | SUPPER. |
|---|---|---|---|
| SUNDAY. | Milk Pottage.<br>Milk, 1-6th of a quart.<br>Oatmeal, 1-16th of a pound.<br>Bread, 1-20th of a quart. loaf. | Beef, roasted, 8 ounces.<br>Potatoes, 12 ounces.<br>Bread, 1-20th of a quart. loaf.<br>Beer, $\frac{1}{2}$ a pint. | Bread, 1-20th of a quart. loaf.<br>Cheese, $1\frac{1}{2}$ oz.<br>Beer, $\frac{1}{2}$ a pint. |
| MONDAY. | Ditto. | Pudding, Suet, $1\frac{1}{2}$ ounce.<br>Flour, 6 ounces.<br>Potatoes, 8 ounces.<br>Beer, $\frac{1}{2}$ a pint. | Bread, 1-20th of a quart. loaf.<br>Milk, $\frac{1}{2}$ a pint. |
| TUESDAY. | Ditto. | Beef, 8 ounces. } stewed.<br>Potatoes, 12 ozs. } stewed.<br>Bread, 1-20th of a quart. loaf.<br>Beer, $\frac{1}{2}$ a pint. | Bread, 1-20th of a quart. loaf.<br>Cheese, $1\frac{1}{2}$ oz.<br>Beer, $\frac{1}{2}$ a pint. |

| | | | |
|---|---|---|---|
| Wednesday. | Milk Pottage.<br>Milk, 1-6th of a quart.<br>Oatmeal, 1-16th of a pound.<br>Bread, 1-20th of a quart. loaf. | Soup, Pease, 1 gill<br>Potatoes, 12 ounces.<br>Bread, 1-20th of a quart. loaf.<br>Beer, ½ a pint. | Bread, 1-20th of a quart. loaf.<br>Milk, ½ a pint. |
| Thursday. | Ditto. | Beef, 8 ounces } stewed.<br>Potatoes, 12 ozs. }<br>Bread, 1-20th of a quart. loaf.<br>Beer, ½ a pint. | Bread, 1-20th of a quart. loaf.<br>Cheese, 1½ oz.<br>Beer, ½ a pint. |
| Friday. | Ditto. | Pudding, Suet, 1½ ounce.<br>Flour, 6 ounces.<br>Potatoes, 8 ounces.<br>Beer, ½ a pint. | Bread, 1-20th of a quart. loaf.<br>Milk, ½ a pint. |
| Saturday. | Ditto. | Mutton, 8 ounces. } stewed.<br>Potatoes, 12 ozs. }<br>Bread, 1-20th of a quart. loaf.<br>Beer, ½ a pint. | Bread, 1-20th of a quart. loaf.<br>Cheese, 1½ oz.<br>Beer, ½ a pint |

N.B. The Meat is estimated as taken from the Butcher, including Bone.
A proportion of the very small Children on six ounces of Meat.

*Perniones*, or chilblains, are very common in the Winter months. They generally first appear about the end of October or beginning of November, and continue to prevail till the end of March. The ulcers formed by the chilblains, are often long in healing, apparently from want of power or tone in the system and a weak circulation, and the scrofulous children suffer most from them.

*Hernia* occasionally appears in some of the children. From the year 1825 to 1841 inclusive, 1,320 children have been admitted into this Institution, (exclusive of those previously admitted and remaining), and during that period only *twenty-two* have had this complaint.

Of this number *twenty* were inguinal, and *two* came down with the testes into the scrotum. *Eighteen* occurred on the right side, *two* on the left, and *two* were on both sides.

Of the total number, *eleven* were cured during their stay in the Institution, *two* died of other complaints, *eight* have been discharged as apprentices to trades, or to their friends, and *one* still remains in the Asylum.

Regarding the age at which the hernia appeared, *seven* occurred between the age of five and nine years, and *fifteen* from nine to fourteen years.

I have rarely been able to trace the immediate cause of rupture in consequence of the children not being aware of it on its first appearance, therefore it is only accidentally discovered, or when they suffer pain from the swelling.

The *Gymnastic Exercises* in use here have been supposed to have a tendency to produce ruptures; without denying this, I cannot say that I have had any case directly traceable to such a cause.

Boys under nine years of age are not permitted to use these exercises. I have tried various kinds of trusses, but have found those made by Mr. Egg, of Piccadilly, answer best.

Among the various diseases which occur here, scrofulous affections form a considerable proportion—such as chronic enlargement, inflammation and suppuration of the cervical and other lymphatic glands; pustular ophthalmia, corneitis, ulcers of the cornea, iritis, &c. Scrofulous affections of the bones, and disease of the elbow, hip and knee joints are common. There has been only one instance of caries of the shoulder-joint, and I believe scrofulous affections of this joint are rare. There have been also several cases of spinal disease.

*Mesenteric Disease* is very frequent. Through the humane consideration of the Commissioners of this Institution, for the health of the children, a certain number afflicted with scrofulous complaints are annually sent to Herne Bay during the Summer months, for the benefit of sea-air and bathing.

Much good results from this—many having returned in a greatly improved state of health, and cured of various scrofulous ulcerations. Others with enlarged lymphatic glands have, in most instances, had them considerably diminished or totally resolved. Some affected with incipient mesenteric disease have received the greatest benefit from their temporary residence at the sea-side, and who, most probably, without this change of air, would have fallen victims to the disease. A few having scrofulous ophthalmia have sometimes been sent, but in these cases no good has ever been derived.

In the preceding statement the amount of disease and mortality may probably appear to be much greater than what occurs in other establishments of children of equal number in this country. I think, however, this may readily be explained, when it is considered that they are admitted at the early age of five years, and consequently a great number of them have to pass through all those diseases which are natural to childhood, and, in addition, that many of them have been born in various climates, East and West Indies, &c. and badly nursed from their earliest infancy.

Sir J. Clark, in his valuable work on the Influence of Climate, makes the following remark—" The great prevalence of pulmonary diseases among the natives of tropical climates who come to this and other cold countries, is doubtless chiefly owing to the influence of a cold and humid atmosphere upon their system. It is in such persons and in *young children* that tuberculous diseases are more speedily induced, and where inflammation appears more intimately connected with the production of tubercles.

The rapid progress of the disease in both classes of persons is to be explained principally, I believe, by the circumstances of their habit of body being that which is most disposed to tuberculous affections,—the most nearly allied to tuberculous cachexy."—I have frequently had occasion to witness the accuracy of this observation in the children admitted here from warm climates.

In conclusion, I wish particularly to observe that, of the 92 deaths specified in the preceding statistical account—53 have died at and under nine years of age, and 39 from ten to fourteen years; and of the whole number, fifty-eight, or nearly two-thirds, have exhibited, on the *post-mortem* examination, a greater or less extent of tubercular disease, whatever might be the proximate cause of death.

It cannot fail to be noticed that pulmonary consumption, and marasmus, are the two most fatal diseases which occur here, and next in fatality—hydrocephalus, a disease which undoubtedly frequently originates from an hereditary and scrofulous taint.

I think, therefore, it is fully shewn, by the foregoing statement, that scrofula and tuberculous cachexy prevail, and are apparently hereditary, in a very great proportion of the children of soldiers. I could have given many more cases from my notes to prove this had I not wished to avoid prolixity and a repetition of cases nearly similar.

A *The manner in which the Diverticulum was attached to the Umbilicus.*
B *The Diverticulum.*
C *Its union with the Ileum.*
D *The upper portion of the Ileum enlarged and extremely vascular.*
E *The lower portion contracted, and in a state approaching Sphacelus.*

RETURN of the NUMBER of SICK TREATED and of FATAL DISEASES in [illegible]

Proportion of SICK to STRENGTH, &c. [illegible]

| Year. | Establishment. | Remained in Hospital 1st January. | Admitted during the Year with Ophthalmia. | Ditto, with Scarlatina. | Ditto, with Rubeola. | Ditto, with Pertussis. | Ditto, with Variola. | Ditto, with Varicella. | Ditto, with Epidemic Catarrh. | Ditto, with Common Fevers. | Ditto, with Porrigo Capitis. | Ditto, with various Cutaneous Diseases. | Ditto, with Chilblains. | Ditto, with Scrofulous Affections of the Bones and Joints. | Ditto, with other Complaints. | Total treated. | Discharged. | Died. |
|---|---|---|---|---|---|---|---|---|---|---|---|---|---|---|---|---|---|---|
| 1825 | 1000 | 80 | 198 | 49 | 83 | 4 | 7 | 7 | .. | 304 | 111 | 224 | 123 | 3 | 348 | 1541 | 1460 | 8 |
| 1826 | 1000 | 73 | 136 | .. | 20 | 12 | .. | 7 | 55 | 171 | 123 | 220 | 106 | 3 | 648 | 1574 | 1505 | 9 |
| 1827 | 1000 | 60 | 249 | 5 | 1 | 2 | 1 | 4 | .. | 105 | 152 | 107 | 127 | 2 | 628 | 1443 | 1386 | 3 |
| 1828 | 1000 | 54 | 165 | 16 | .. | 2 | .. | 10 | .. | 101 | 158 | 245 | 85 | 1 | 654 | 1491 | 1417 | 14 |
| 1829 | 850 | 60 | 96 | .. | .. | .. | 2 | 5 | .. | 42 | 118 | 116 | 220 | 4 | 642 | 1305 | 1247 | 7 |
| 1830 | 800 | 51 | 107 | .. | 48 | .. | 5 | 10 | .. | 66 | 122 | 58 | 201 | 1 | 782 | 1451 | 1393 | 7 |
| 1831 | 800 | 51 | 81 | .. | .. | 23 | .. | 1 | .. | 136 | 113 | 69 | 154 | 3 | 717 | 1348 | 1288 | 8 |
| 1832 | 700 | 52 | 60 | .. | .. | 3 | 1 | .. | .. | 35 | 94 | 40 | 130 | 3 | 794 | 1212 | 1167 | 9 |
| 1833 | 600 | 36 | 59 | .. | .. | 5 | 2 | 2 | 140 | 23 | 85 | 37 | 151 | .. | 603 | 1143 | 1106 | 3 |
| 1834 | 450 | 34 | 69 | 38 | 48 | .. | .. | .. | .. | 79 | 60 | 29 | 69 | 2 | 605 | 1033 | 1006 | 3 |
| 1835 | 350 | 24 | 80 | 10 | 1 | 1 | .. | 2 | .. | 23 | 34 | 52 | 102 | 1 | 375 | 705 | 672 | 5 |
| 1836 | 350 | 28 | 57 | 1 | .. | 1 | 3 | 3 | .. | 31 | 22 | 44 | 76 | .. | 369 | 635 | 614 | 1 |
| 1837 | 350 | 20 | 30 | .. | 4 | .. | .. | 1 | 60 | 32 | 45 | 261 | 72 | 1 | 277 | 803 | 769 | 3 |
| 1838 | 350 | 31 | 20 | 11 | .. | 2 | 1 | 3 | .. | 38 | 24 | 140 | 64 | .. | 303 | 637 | 607 | 2 |
| 1839 | 350 | 27 | 37 | 7 | 35 | .. | .. | .. | .. | 45 | 79 | 185 | 47 | .. | 215 | 677 | 638 | 5 |
| 1840 | 350 | 34 | 38 | 2 | .. | 10 | 1 | 5 | .. | 26 | 112 | 176 | 40 | 5 | 244 | 693 | 659 | 3 |
| 1841 | 400 | 31 | 27 | .. | .. | 4 | .. | .. | .. | 85 | 113 | 73 | 32 | 1 | 245 | 611 | 588 | 2 |
| Total | | 746 | 1509 | 139 | 240 | 69 | 23 | 60 | 255 | 1342 | 1565 | 2076 | 1799 | 30* | 8449 | 18302 | 17522 | 92 |

* N.B. This Total includes—

Disease of the Spine................ [illegible]

Ditto of the Inferior Maxillary Bone [illegible]

Ditto of the Scapula............. [illegible]

Ditto of the Rib ................. [illegible]

Ditto of the Shoulder-joint........ [illegible]

Ditto of the Elbow-joint .......... [illegible]

Hospital of the Royal Military Asylum at Chelsea, with the Average
From the 1st January, 1825, to 31st December, 1841, inclusive.

| Average daily number of Sick in Hospital throughout the Year. | Average Proportion of Sick to Average Strength. | Average Proportion of Deaths to total Number of Sick treated. | Proportion of Deaths to Average Strength. | Diseases which have proved fatal in each Year. | | | | | | | | | | | | | | | | | | | | |
|---|---|---|---|---|---|---|---|---|---|---|---|---|---|---|---|---|---|---|---|---|---|---|---|---|
| | | | | Phthisis Pulmonalis. | Pneumonia. | Hæmoptysis. | Carditis. | Pertussis. | Cynanche Trachealis. | Enteritis. | Peritonitis. | Hydrocephalus. | Scarlatina Maligna. | Rubeola. | Ascites and Anasarca. | Tabes Mesenterica. | Disease of the Hip-joint. | Disease of the Spine. | Purpura Hæmorrhagica. | Icterus. | Febris Typhus. | Scrofulous Abscess of both Kidneys. | Fracture of the Skull. | Abscess in the Brain. |
| 71 | 1 in $13\frac{7}{10}$ | 1 in 192 | 1 in 122 | 1 | | | | | | | 1 | 1 | | 3 | | 2 | | | | | | | | |
| 65 | 1 ,, $14\frac{7}{8}$ | 1 .. 175 | 1 .. 108 | 2 | 1 | | | 3 | | | | 1 | | 2 | | | | | | | | | | |
| 62 | 1 ,, $15\frac{5}{7}$ | 1 .. 481 | 1 .. 325 | | 1 | | | | | | | | | | | 2 | | | | | | | | |
| 60 | 1 ,, $15\frac{5}{6}$ | 1 .. 106 | 1 .. 68 | 2 | | 1 | 1 | | | | | 5 | | | 1 | 1 | | 1 | 1 | | 1 | | | |
| 53 | 1 ,, $15\frac{1}{5}$ | 1 .. 186 | 1 .. 115 | 3 | 1 | | | | | 1 | | | | | | 1 | | | | 1 | | | | |
| 48 | 1 ,, $16\frac{1}{5}$ | 1 .. 207 | 1 .. 111 | | 1 | 1 | | | | 1 | | 1 | | | | 2 | 1 | | | | | | | |
| 43 | 1 ,, $16\frac{1}{3}$ | 1 .. 168 | 1 .. 87 | 2 | | | 1 | 1 | | | | | | | | 1 | 2 | | | | | | | 1 |
| 36 | 1 ,, 17 | 1 .. 134 | 1 .. 67 | 3 | 1 | | 1 | | | | | 2 | | | | 2 | | | | | | | | |
| 35 | 1 ,, $14\frac{1}{2}$ | 1 .. 381 | 1 .. 168 | | | | | | | 1 | | 1 | | | | | | 1 | | | | | | |
| 32 | 1 ,, $12\frac{3}{4}$ | 1 .. 334 | 1 .. 136 | 1 | | | | | | 1 | | | 1 | | | | | | | | | | | |
| 21 | 1 ,, $15\frac{1}{3}$ | 1 .. 141 | 1 .. 64 | 1 | | | | | 1 | | | 1 | | | | 2 | | | | | | | | |
| 18 | 1 ,, $15\frac{1}{2}$ | 1 .. 635 | 1 .. 279 | | | | | | | | | | | | | | | | | | | | 1 | |
| 24 | 1 ,, $11\frac{5}{8}$ | 1 .. 267 | 1 .. 93 | 2 | | | | | | | | | | | | 1 | | | | | | | | |
| 23 | 1 ,, $12\frac{2}{3}$ | 1 .. 318 | 1 .. 146 | | | | | | | | | 1 | | | 1 | | | | | | | | | |
| 25 | 1 ,, $12\frac{1}{2}$ | 1 .. 135 | 1 .. 65 | 2 | | | | | | | | | | | | 2 | | | | | | 1 | | |
| 28 | 1 ,, 12 | 1 .. 231 | 1 .. 112 | | | | 1 | 1 | | | | | | | | 1 | | | | | | | | |
| 26 | 1 ,, $14\frac{7}{13}$ | 1 .. 305 | 1 .. 189 | 1 | | 1 | | | | | | | | | | | | | | | | | | |
| | | | | 20 | 5 | 3 | 4 | 5 | 1 | 4 | 1 | 13 | 1 | 5 | 2 | 17 | 3 | 2 | 1 | 1 | 1 | 1 | 1 | 1 |

Disease of the Finger-joint ............. 1

Ditto of the Hip-joint ............... 4

Ditto of the Knee-joint .............. 1

Ditto of the Ankle-joint... ........... 3

Ditto of the Tibia and Fibula ...... .. 1

Ditto of the Metatarsal Bones ........ 4

*Tabular Return of Boys who have had Small-pox in the Royal Military Asylum, Chelsea, from Jan.* 1825, *to Dec.* 1841.

| No. | Names. | Date of admission into the Asylum. | On admission reported to have had, | When had small-pox in the Asylum. | Age when attacked with small-pox | REMARKS. |
|---|---|---|---|---|---|---|
| 1 | Jno. Law . . . . . . | Oct. 1820 | Cow-pox | March, 1825 | 10 yrs. | Had a severe form of the disease, leaving numerous marks. |
| 2 | Edwd. Hill . . . . . . | June, 1823 | ditto | April, 1825 | 10 .. | Had a mild disease. |
| 3 | Alex. Leslie . . . . . . | Feb. 1825 | ditto | April, 1825 | 10 .. | Ditto. |
| 4 | Frs. Mc Manus .. | May, 1819 | Small-pox | May, 1825 | 12 .. | Ditto. |
| 5 | Wm. Wilson . . . . | Aug. 1821 | ditto | May, 1825 | 12 .. | Ditto. |
| 6 | Frs. Gritton . . . . . . | Oct. 1823 | Cow-pox | June, 1825 | 11 .. | Ditto. |
| 7 | Jno. Mc Ilhatton.. | Oct. 1824 | Small-pox | Aug. 1825 | 10 .. | Ditto. |
| 8 | George Neil . . . . . . | Nov. 1827 | Cow-pox | Dec. 1827 | 6 .. | Had a severe and confluent form of the disease, leaving numerous marks, and followed by phlegmonous abscesses. |
| 9 | Jno. Gowday . . . . | June, 1826 | ditto | Feb. 1829 | 9 .. | Ditto, leaving numerous marks, and followed by abscesses on the scalp. |
| 10 | Owen Mulhearn .. | May, 1827 | ditto | Nov. 1829 | 12 .. | Had a mild disease. |
| 11 | Jno Rose . . . . . . . . | Feb. 1826 | ditto | March, 1830 | 13 .. | Ditto. |
| 12 | Wm. Hassell . . . . | March, 1827 | ditto | Aug. 1830 | 13 .. | Ditto. |
| 13 | Sam. Hunter . . . . | April, 1825 | ditto | Oct. 1830 | 11 .. | Had a severe disease, leaving a few marks. |
| 14 | Peter Loughrea .. | Feb. 1829 | Small-pox | Oct. 1830 | 11 .. | Had a severe and confluent form of the disease, leaving numerous marks. |
| 15 | Dan. Weir . . . . . . | Jan. 1825 | Cow-pox | Oct. 1830 | 13 .. | Had a mild disease. |
| 16 | Jno. Flannagan .. | March, 1830 | ditto | April, 1832 | 8 .. | Ditto. |
| 17 | Wm. Foster . . . . . . | Aug. 1830 | ditto | Feb. 1833 | 11 .. | Ditto. |
| 18 | Wm. Salmon . . . . | June, 1826 | ditto | March, 1833 | 13 .. | Ditto. |
| 19 | James Froome . . . . | March, 1832 | ditto | May, 1836 | 13 .. | Had a confluent form of the disease, leaving permanent marks. |
| 20 | James Davie . . . . . . | April, 1832 | ditto | May, 1836 | 13 .. | Had a mild disease. |
| 21 | Wm. Miller . . . . . . | Aug. 1830 | ditto | June, 1836 | 13 .. | Had a severe and confluent form of the disease, leaving numerous marks. |
| 22 | Chas. Mason . . . . | May, 1832 | ditto | June, 1838 | 13 .. | Ditto. |
| 23 | Thos. Little . . . . . . | Aug. 1837 | ditto | Nov. 1840 | 10 .. | Had a mild disease. |

## Figures and Tables

**Figure f1:**